# Chitin Recognition via Chitotriosidase Promotes Pathologic Type-2 Helper T Cell Responses to Cryptococcal Infection

**DOI:** 10.1371/journal.ppat.1004701

**Published:** 2015-03-12

**Authors:** Darin L. Wiesner, Charles A. Specht, Chrono K. Lee, Kyle D. Smith, Liliane Mukaremera, S. Thera Lee, Chun G. Lee, Jack A. Elias, Judith N. Nielsen, David R. Boulware, Paul R. Bohjanen, Marc K. Jenkins, Stuart M. Levitz, Kirsten Nielsen

**Affiliations:** 1 Department of Microbiology, Medical School, University of Minnesota, Minneapolis, Minnesota; 2 Division of Infectious Diseases and Immunology, Department of Medicine, University of Massachusetts Medical School, Worcester, Massachusetts; 3 Section of Pulmonary and Critical Care Medicine, Department of Internal Medicine, Yale University School of Medicine, New Haven, Connecticut; 4 Warren Alpert Medical School, Division of Biology and Medicine, Brown University, Providence, Rhode Island; 5 Department of Pathology and Laboratory Medicine, School of Medicine, University of North Carolina, Chapel Hill, Chapel Hill, North Carolina; 6 Division of Infectious Diseases and International Medicine, Medical School, University of Minnesota, Minneapolis, Minnesota; University of California San Francisco, UNITED STATES

## Abstract

Pulmonary mycoses are often associated with type-2 helper T (Th2) cell responses. However, mechanisms of Th2 cell accumulation are multifactorial and incompletely known. To investigate Th2 cell responses to pulmonary fungal infection, we developed a peptide-MHCII tetramer to track antigen-specific CD4+ T cells produced in response to infection with the fungal pathogen *Cryptococcus neoformans*. We noted massive accruement of pathologic cryptococcal antigen-specific Th2 cells in the lungs following infection that was coordinated by lung-resident CD11b+ IRF4-dependent conventional dendritic cells. Other researchers have demonstrated that this dendritic cell subset is also capable of priming protective Th17 cell responses to another pulmonary fungal infection, *Aspergillus fumigatus*. Thus, higher order detection of specific features of fungal infection by these dendritic cells must direct Th2 cell lineage commitment. Since chitin-containing parasites commonly elicit Th2 responses, we hypothesized that recognition of fungal chitin is an important determinant of Th2 cell-mediated mycosis. Using *C. neoformans* mutants or purified chitin, we found that chitin abundance impacted Th2 cell accumulation and disease. Importantly, we determined Th2 cell induction depended on cleavage of chitin via the mammalian chitinase, chitotriosidase, an enzyme that was also prevalent in humans experiencing overt cryptococcosis. The data presented herein offers a new perspective on fungal disease susceptibility, whereby chitin recognition via chitotriosidase leads to the initiation of harmful Th2 cell differentiation by CD11b+ conventional dendritic cells in response to pulmonary fungal infection.

## Author Summary

Humans often inhale potentially pathogenic fungi in the environment. While CD4+ helper T (Th) cells are required for protection against invasive disease, a subset of Th cells, called Th2 cells, are associated with increased mortality and allergy/asthma morbidity. Our study aimed to unravel the cellular and molecular basis of pulmonary Th2 cell induction in response to lethal infection with *Cryptococcus neoformans*. Antigen-presenting cells coordinate naïve Th cell priming and differentiation, but the precise leukocyte responsible for Th2 cell expansion to pulmonary cryptococcal infection has not been determined. Using an experimental mouse model of pulmonary cryptococcosis, we show that a subset of lung-resident dendritic cells is uniquely required for Th2 cell induction. We additionally sought to identify the molecular signal received by the host that allows dendritic cells to selectively induce Th2 cells. Since parasites and fungi elicit Th2 cell responses and both produce chitin, a molecule not found in vertebrates, we hypothesized that recognition of fungal chitin is a determinant of fungal disease. Here, we demonstrate that *C. neoformans* chitin and the host-derived chitinase, chitotriosidase, promote Th2 cell accumulation and disease. These findings highlight a promising target of next generation therapies aimed at limiting immunopathology caused by pulmonary fungal infection.

## Introduction

Pulmonary mycoses, ranging from invasive fungal infection to severe asthma with fungal sensitization, affect millions of people worldwide [1,2]. Fungi inhabit a multitude of ecological niches, and consequently, humans continuously encounter potentially pathogenic fungi in the environment. Subsequent disease is determined by the size of the innoculum, virulence of the microbe, and immune status of the host. In particular, CD4+ helper T (Th) cell subsets are critical mediators of the immune response to fungal exposure. Interferon-γ from Th1 cells and interleukin (IL)-17 from Th17 cells contribute to protective immunity via classical activation of macrophages and neutrophil recruitment, respectively [3]. Conversely, Th2 cell production of IL-4, IL-5, and IL-13 impels eosinophilia, alternative macrophage activation, mucus and IgE production, and airway obstruction [4]. These type-2 responses drive fungal-associated allergies and positively correlate with invasive fungal disease severity [4]. Although a fair amount is known about type-2 responses and their downstream consequences, the basis of Th2 cell induction associated with pulmonary mycosis is less well defined.

Antigen presentation by an immune cell bearing major histocompatibility II (MHCII) is required for naïve Th cell priming and differentiation. Thus, a cellular intermediate must coordinate Th2 cell induction. Professional antigen presenting cells direct Th cell fate, and inflamed lungs contain several ontologically distinct immune cells with this potential capability [5]. The precise leukocyte subset responsible for priming Th2 cells, as well as the location that this event occurs, whether at the site of infection or within secondary lymphoid tissue, has not been comprehensively investigated. Furthermore, specific features of the infection that lead to Th2 cell lineage commitment remain largely unexplored in the context of pulmonary fungal infection.

While some models attribute induction of type-2 responses to protease cleavage of host proteins and wound repair of lung injury [6], many microbes that elicit Th2 cell responses produce chitin [7]. Chitin is a polysaccharide composed of polymeric *N*-acetylglucosamine. The rigidity of chitin is utilized in the cell wall of fungi as well as the exoskeleton of arthropods and filarial sheath of parasitic worms. Higher organisms rely on keratin for similar structural purposes, and as a result, vertebrates do not synthesize or store chitin. These differences allow an opportunity for the vertebrate immune system to detect chitin-containing pathogens as foreign [8,9]. While chitin detection may prove beneficial to the host in the context of parasitic infection [10], we hypothesize that inappropriate or dysregulated Th2 responses instigated by recognition of chitin promotes fungal pathogenesis.

Chitinases are a pivotal component of the host response to chitinous organisms [11]. Chitin is positioned beneath layers of mannans and glucans in the fungal cell wall, thus secreted host chitinases are needed to penetrate the wall matrix and make chitin fragments available to host surveillance [12]. Mammals encode two functional chitinases, chitotriosidase (Chit1) and acidic mammalian chitinase (AMCase) [11]. A naturally occurring allele of *CHIT1* renders the enzyme inactive [13], and these mutations have been associated with susceptibility to parasitic worm infection in humans [14]. Likewise, AMCase has been linked to eosinophilia [15] and alternative macrophage activation [16] in mouse models of pulmonary allergy. Consequently, we reasoned that mammalian chitinases could be necessary for efficient host recognition of fungal chitin and subsequent Th2 cell priming.

Using an inhalation model of *Cryptococcus neoformans* infection and novel reagents to detect *Cryptococcus*-specific Th cells, we unravel the basis for Th2 cell induction in response to pulmonary fungal infection. We report profound accumulation of detrimental Th2 cells in the lungs of infected mice. We additionally show that lung-resident CD11b+ interferon regulatory factor (IRF) 4-dependent conventional dendritic cells present antigen to Th cells and drive potent Th2 differentiation at the site of infection in the lungs. Surprisingly, our results demonstrate that an excess of fungal chitin, as well as digestion of chitin via Chit1, and not AMCase, lead to chitin detection, Th2 cell accumulation, and enhanced disease. Lastly, we observed increased Chit1 activity in humans with confirmed fungal infections, reinforcing the relevance of Chit1 in human disease. This study offers novel insights into the cellular source of antigen presentation and molecular basis of chitin recognition via Chit1 that underlies deleterious Th2 cell formation during pulmonary mycosis.

## Results

### Pulmonary cryptococcal infection results in profound accumulation of antigen-specific Th2 cells

**Fig 1:**
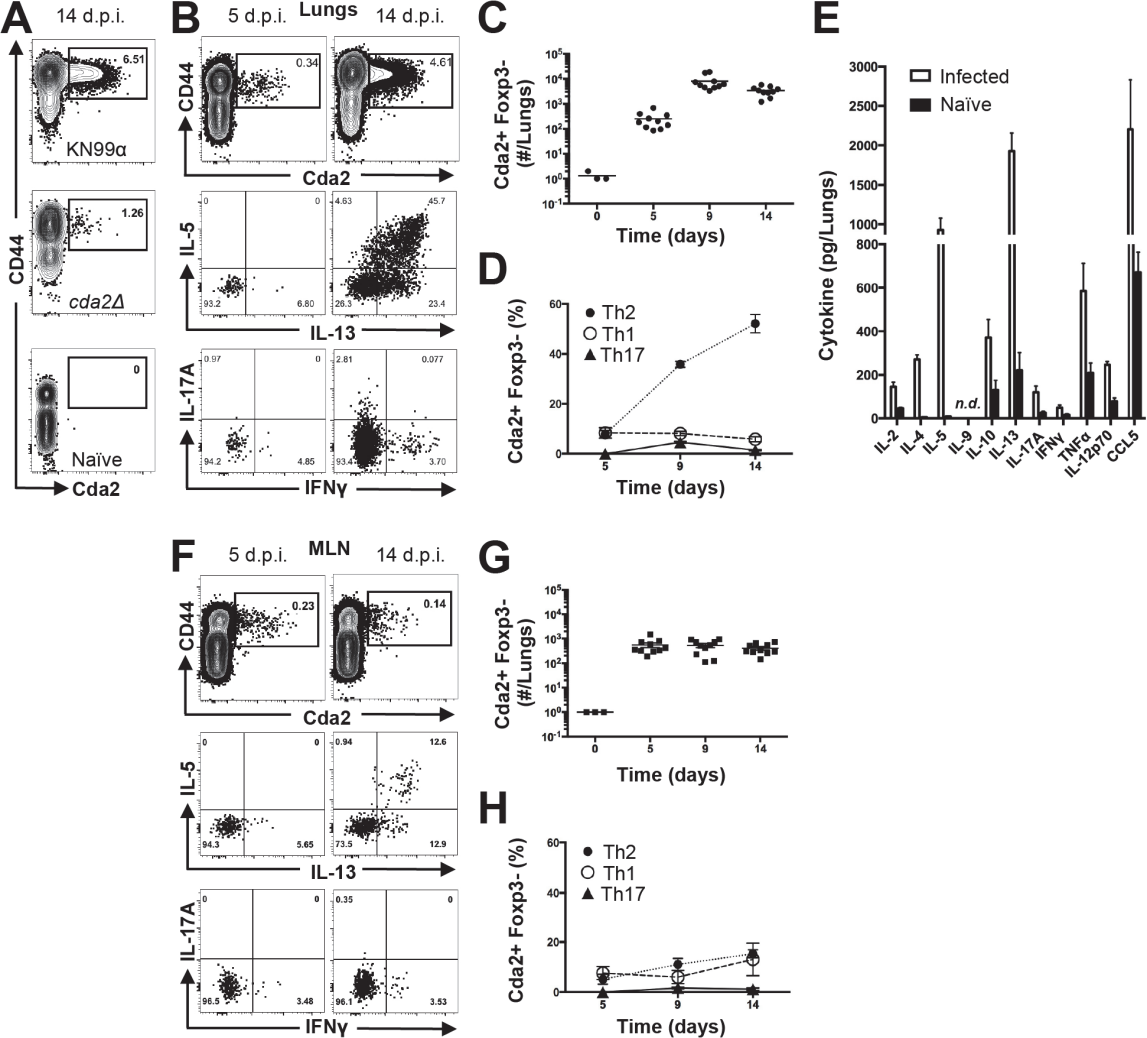
Type-2 Helper T Cells Accumulate in the Lungs of Mice Infected with *C. neoformans*. (**A**) Cda2-MHCII tetramer identifies *C. neoformans*-specific helper T (Th) cells from mice 14 days post-infection with strain KN99α, *cda2Δ*, or age-matched, naïve mice. Flow cytometry plot (**B**) or graphs (**C&D**) from lung digests showing CD4+, Foxp3-, CD44+ Cda2+ Th cells expressing Th1 (IFNγ), Th2 (IL-5 & IL-13), or Th17 (IL-17A) cytokines. (**E**) Cytokines from lung homogenates 14 days post-infection with KN99α or age-matched, naïve mice. Flow cytometry plot (**F**) and graphs (**G&H**) from mediastinal lymph node suspensions of Th cells expressing Th1, Th2, or Th17 cytokines. Data are presented as mean +/- standard error with 2 independent experiments of at least 5 mice per group. Cda = Chitin deacetylase, CCL = chemokine ligand, IFN = interferon, IL = interleukin, MLN = mediastinal lymph node, TNF = tumor necrosis factor.

We utilized a murine model of pulmonary cryptococcosis to investigate Th cell priming during fungal infection. Upon inhalation, *Cryptococcus neoformans* establishes a robust lower respiratory tract infection that causes tissue damage and ultimately leads to mortality from pulmonary complications and dissemination resulting in meningoencephalitis. To distinguish Th cell responses to infection from non-specific wound healing Th2 cell responses, we generated a recombinant peptide-major histocompatibility class II (pMHCII) tetramer that enabled identification of *C. neoformans* antigen-specific Th cells. The pMHCII tetramer contains a 13 amino acid peptide from an immunodominant cryptococcal protein, chitin deacetylase 2 (Cda2) (Table 1) [17]. The Cda2-MHCII tetramer labeled a population of antigen-experienced (i.e. CD44+) Th cells, but it did not stain non-activated (i.e. CD44−) Th cells from *C. neoformans* infected mice or CD44+ Th cells from naive mice (Figs. 1A, S1 for flow cytometry gating). In addition, mice infected with a *C. neoformans* mutant (*cda2Δ*) that lacks Cda2 protein expression [18] had marked reductions in Cda2-MHCII tetramer binding cells (Fig. 1A). Though Cda2 contains a dominant CD4+ T cell epitope, cross-reactivity to other closely related cryptococcal proteins likely account for the remaining tetramer binding Th cells generated during infection with *cda2Δ* (Table 1). Taken together, these studies show the Cda2-MHCII tetramer reliably identified antigen-specific CD4+ T cells produced in response to *C. neoformans* infection.

**Table 1:** CD4+ T cell Cda2-MHCII tetramer peptide sequence and putative cross reactive peptides from other *C. neoformans* chitin deacetylases.

Gene	Amino Acid Sequence^a^	MHCII Score^b^
***cda1***	WSHP**ALTTLTNEE**IVAELG	36
***cda2^c^***	WSHQ**YMTALSNEV**VFAELY	74
***cda3***	WSHP**YMTTLTNEQ**VVGELG	56
***fpd1***	WSHA**DLTQLDESG**INDELS	13

The **bold** sequences indicate regions that align with the P1-P9 residues within the Cda2-MHCII tetramer core. The underlined sequences correspond to the total peptide included in the Cda2-MHCII tetramer.

^a^ Homologous amino acid sequences within the catalytic domain of chitin deacetylases.

^b^ Peptide loading score on MHCII I-A^B^ (74).

^c^ Cda2-MHCII tetramer made from this gene and amino acid sequence.

We characterized the immune response in the lung and lung-draining mediastinal lymph node (MLN) to determine the relative contributions of each site to CD4+ T cell subset differentiation. Pulmonary cryptococcal infection resulted in a progressive accumulation of Cda2-MHCII-specific T cells in the lungs that predominately expressed the Th2 cytokines IL-5 and/or IL-13 (Fig. 1B-D). In addition, Th2 cytokines IL-5, IL-13, and CCL5 were among the most abundant cytokines present in infected lung homogenates (Fig. 1E), and eosinophils, a downstream correlate of type-2 cytokines, represented an overwhelming majority of the bulk leukocyte population in the lungs (S2 Fig. for flow cytometry gating, S3A Fig.). In contrast, the Cda2-MHCII-specific Th2 cell response within the MLN (Fig. 1F-H) was significantly lower than the response observed in the lungs, and eosinophils comprised an insubstantial component of the lymph node resident leukocytes (S3 Fig.). These findings collectively suggest the local inflammatory environment in the lung may shape the differentiation and/or promote the selective expansion of Th2 cells.

### Interleukin-2 cytokine/antibody complexes enhance Th2 cell expansion and disease

**Fig 2:**
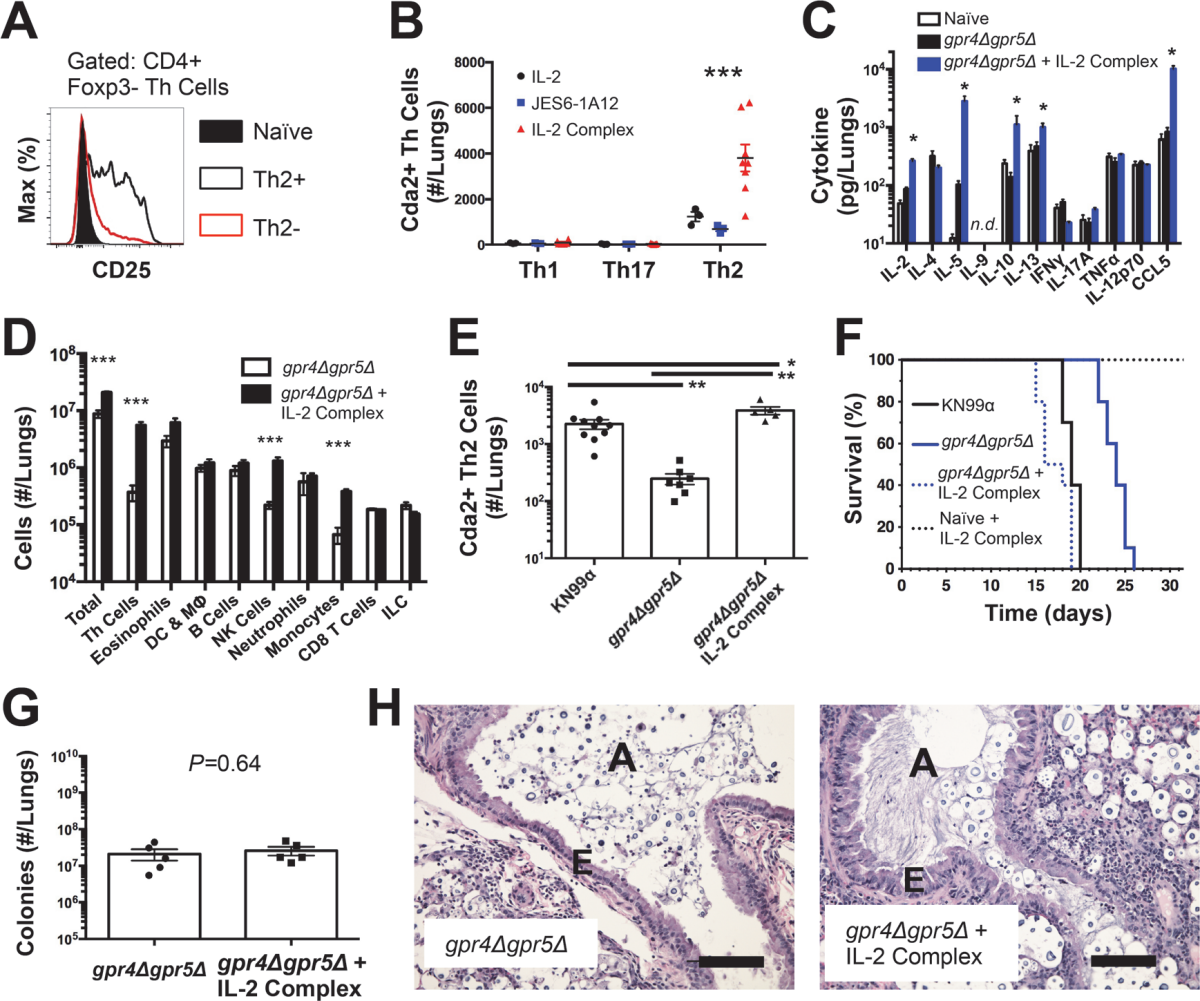
IL-2 Complexes Augment Type-2 Helper T Cells and Enhance Fungal Disease. (**A**) CD25 expression by naïve (CD4+, CD44−), Th2- (CD4+, CD44+, IL-5-, IL-13−), and Th2+ (CD4+, CD44+, IL-5+, IL-13+) cells. (**B**) Cda+ Th cell numbers in the lungs of *gpr4Δgpr5Δ* infected mice treated with 5 μg IL-2, 25 μg anti-IL-2 antibody (JES6-1A12), or both (IL-2 Complex). Th1, Th17, and Th2 cells were identified by production of IFNγ, IL-17A, and IL-5/13, respectively. (**C**) Cytokines from lung homogenates of mice infected with *gpr4Δgpr5Δ* or age-matched naïve animals, with or without IL-2 complex treatment. (**D**) Pulmonary leukocytes from mice infected with *gpr4Δgpr5Δ*, with or without IL-2 complex treatment. (**E**) Cda2+ Th2 cells from lungs of mice infected with fully virulent KN99α, attenuated *gpr4Δgpr5Δ*, or mice infected with *gpr4Δgpr5Δ* and treated with IL-2 complex. (**F**) Survival of naïve mice or mice infected with *gpr4Δgpr5Δ* either with or without IL-2 complex treatment. *P*-value represents log-rank test comparing each survival curve with 10 mice per group to: *gpr4Δgpr5Δ* vs. *gpr4Δgpr5Δ* + IL-2 complex, *P*<0.0005; *gpr4Δgpr5Δ* vs. KN99α, *P*<0.0005; *gpr4Δgpr5Δ* + IL-2 complex vs. KN99α, *P* = 0.23. (**G**) Fungal burden within lungs of mice with or without IL-2 complex 14 days post-infection with *gpr4Δgpr5Δ*. (**H**) Hematoxylin and eosin staining of lungs from mice infected with *gpr4Δgpr5Δ* or similarly infected and treated with IL-2 complexes. A = brochial airway, E = epithelial cell layer. Data are presented as the mean +/- standard error with at least 2 independent experiments per group. * = *P* < 0.05, ** = *P* < 0.005, *** = *P* < 0.0005 by Mann-Whitney *U*. Cda = chitin deacetylase, CCL = chemokine ligand, DC = Dendritic Cells, IFN = interferon, IL = interleukin, ILC = Innate Lymphoid Cells, MΦ = Macrophages, NK = Natural Killer. TNF = tumor necrosis factor.

Due to the seemingly contradictory roles of Th2 cells in beneficial wound healing responses and harmful allergic disease [19], it is not entirely clear whether Th2 cells simply correlate with or cause disease associated with fungal infection. To test the causal relationship of Th2 cells with disease severity in this model, we augmented the endogenous Th2 cell response to fungal infection using IL-2 cytokine/antibody complex treatment. IL-2 can be targeted to the high affinity IL-2 receptor to enhance Th cell proliferation by conjugating IL-2 cytokine with anti-IL-2 antibody to form IL-2 cytokine/antibody complexes [20,21]. Since Th2 cells generated during pulmonary cryptococcal infection expressed high levels of the alpha chain of the high affinity IL-2 receptor (CD25) (Fig. 2A), we sought to use IL-2 complexes to boost the Th2 cell response.

The wildtype strain of *C. neoformans*, KN99α, induces an extremely aggressive infection that leaves little room to increase the Th2 cell response. Consequently, we used an attenuated strain of *C. neoformans, gpr4Δgpr5Δ* (attenuation explained below/Fig. 5; deficient in production of large, chitinous cells). Treatment with IL-2 complexes increased Th2 cell numbers compared with similarly infected mice receiving antibody or cytokine alone (Fig. 2B). Th2 cells and cytokines were also elevated in lung homogenates from infected mice treated with IL-2 complexes (Fig. 2C&D). In addition to Th2 cells, Regulatory T (Treg) cells can be expanded by IL-2 complex treatment [20,21] (S4A Fig.). This increase of Treg cells in mice treated with the IL-2 complex could theoretically suppress protective Th cell responses and allow Th2 cells to predominate the response. However, IL-2 complex treatment did not affect Th1 or Th17 cell numbers (Fig. 2B) and only minimal changes in IFNγ cytokine (Fig. 2C) and monocyte accumulation (Fig. 2D) were observed, showing IL-2 complex treatment did not eliminate the effector activity of protective Th1 cells (Fig. 2D). Instead, Schulze et al. [22] showed Treg cells suppress Th2 cells during cryptococcal infection (S4B–S4C Fig.), suggesting the increase in Treg cells due to IL-2 complex treatment would actually limit Th2 cell accumulation in this system. Hence, IL-2 complexes can be used to augment the Th2 cell response during pulmonary fungal infection and assess the relationship between Th2 cells and fungal disease.

If Th2 cells promote disease, we hypothesized that increasing the Th2 response should accelerate death during infection with the virulence-attenuated strain, *gpr4Δgpr5Δ*. IL-2 complex treatment increased the Th2 cell response to levels even higher than the fully virulent KN99α infection (Fig. 2E). Treatment with IL-2 complexes also greatly reduced the survival time of infected mice (Fig. 2F) without affecting pulmonary fungal burden (Fig. 2G). Uninfected mice treated with the same regimen of IL-2 complexes survived more than 30 days and remained healthy (Fig. 2F), indicating the IL-2 complex treatment targeted detrimental cells that were only present during infection. IL-2 treatment of infected mice also induced obvious lung pathology consistent with increased Th2 activity, noted by increased metaplasia of the bronchiolar epithelium and mucous obstruction of the airways (Fig. 2H). En masse, these data indicate that Th2 cells exacerbate pulmonary disease during fungal infection.

### Th2 cells are primed at the site of infection by IRF4-dependent conventional dendritic cells

**Fig 3:**
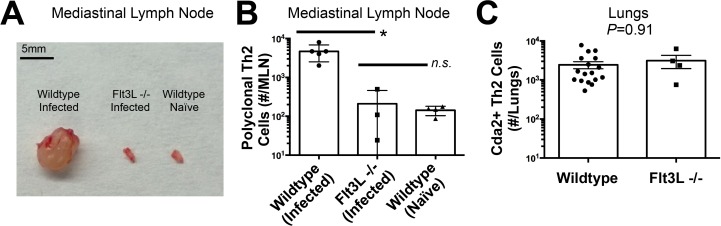
Lymphoid Priming is Dispensible for Pulmonary Th2 Cell Induction during Cryptococcal Infection. (**A**) Lymphadenopathy of mediastinal lymph nodes (MLN) after 14 days post-infection with strain KN99α. (**B**) Quantification of IL-5+ IL-13+ polyclonal Th2 cells from the MLN in wild-type and Flt3L−/− mice. (**C**) Quantification of IL-5+ IL-13+ antigen-specific Th2 cells contained in the lungs of wild-type and Flt3L−/− mice.

The diminished Th2 response in the MLN compared to the lung led us to question whether lymphoid priming was required for Th2 cell induction during pulmonary fungal infection. Fms-like tyrosine kinase 3 ligand (Flt3L) is a differentiation factor for several hematopoietic cell subsets, and genetic deletion of Flt3L causes defects in antigen presenting cell traffic between the site of infection and secondary lymphoid organs [23]. Flt3L deficient mice infected with *C. neoformans* neither experienced mediastinal lymphadenopathy (Fig. 3A) nor elicited a polyclonal Th2 response in the MLN (Fig. 3B). Surprisingly, the Th2 cell response in the lungs after *C. neoformans* infection was unaffected by Flt3L deficiency compared to wildtype animals (Fig. 3C), indicating lymphoid priming is not required for pulmonary Th2 cell accumulation.

**Fig 4:**
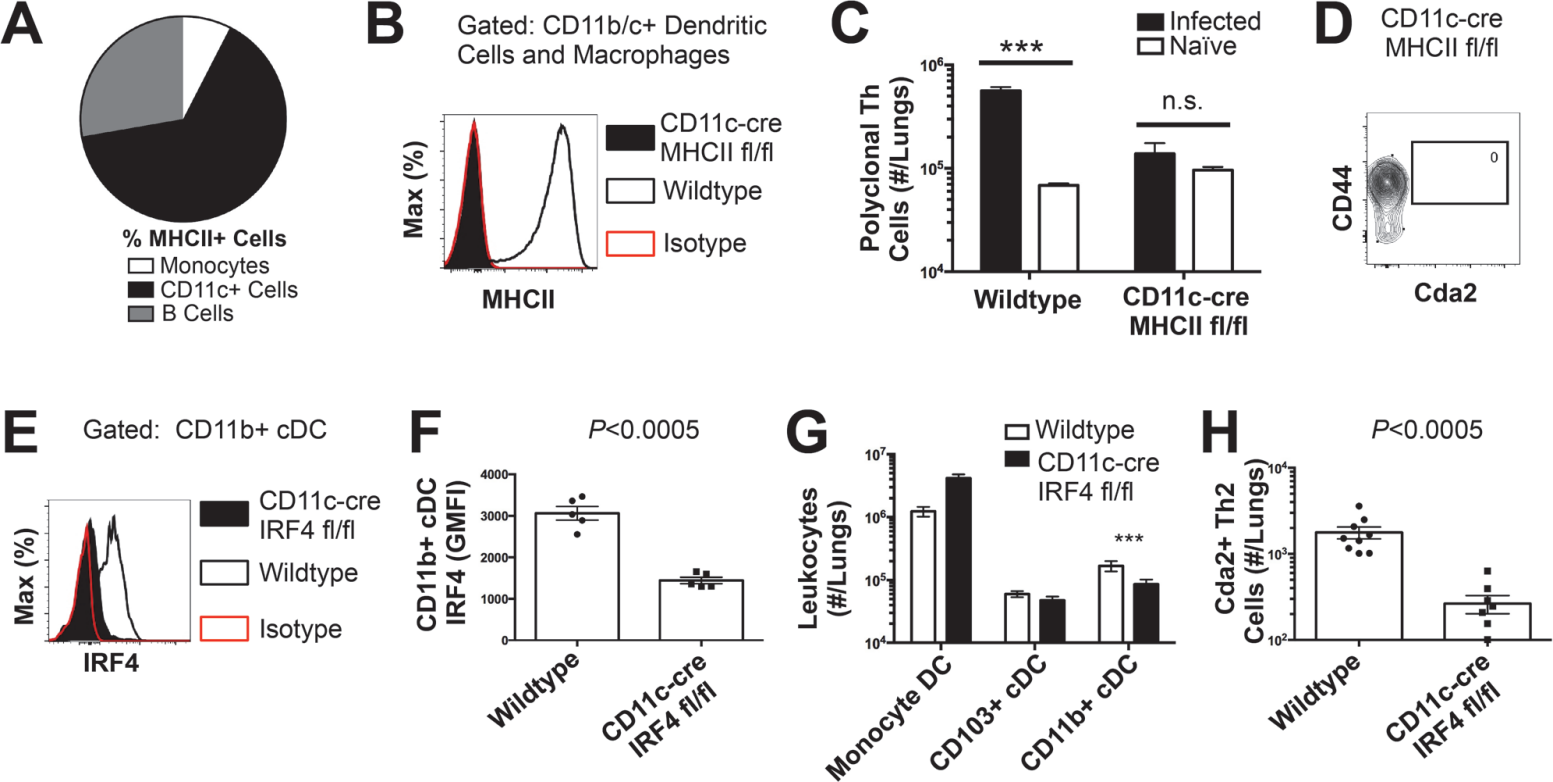
Interferon Regulatory Factor 4-Dependent Conventional Dendritic Cells Coordinate Th2 Cell Induction. **(A)** Antigen-presenting cells as a proportion of total MHCII+ cells in the lungs of infected mice. **(B)** MHCII expression by CD11b/c+ cells from mice 14 days post-infection with KN99α. Isotype refers to CD11b/c+ cells from wildtype mice stained with a rat IgG2b antibody of irrelevant specificity. **(C)** Quantification of pulmonary CD4+ Foxp3- CD44+ pulmonary Th cells. **(D)** Biexponential plot of CD4+ Foxp3- cells, indicating the absence of antigen-specific (CD44−, Cda2−) cells in the lungs of CD11c-cre MHCII mice infected with KN99α. **(E)** IRF4 expression by CD11b+ cDC from lungs of CD11cre IRF4 fl/fl or wildtype mice 14 days post-infection with KN99α. Isotype refers to CD11b+ cDC from wildtype mice stained with a rat IgG1 antibody of irrelevant specificity. **(F)** Geometric mean fluorescence intensity of CD11b+ cDC in from the lungs of infected CD11c-re IRF4 fl/fl mice. **(G)** Quantification of monocytes and dendritic cell subsets from the lungs of mutant or wildtype mice infected with KN99α. **(H)** Quantification of IL-5+ IL-13+ antigen-specific Th2 cells contained in the lungs of mutant or wildtype mice 14 days post-infection with KN99α. Data are presented as mean +/- standard error. * = *P* < 0.05, *** = *P* < 0.0005, and n.s. = not significant by Mann-Whitney *U*. Cda = chitin deacetylase, Flt3L = fms-like tyrosine kinase 3 ligand, MHC = major histocompatibility complex, cDC = conventional dendritic cells, IRF4 = interferon regulatory factor 4.

To determine the immune cell intermediate that primes Th2 cells in the lungs, we relied on the fact that Th cells are MHCII restricted. Three leukocyte subsets that express MHCII exist in the lungs of mice infected with *C. neoformans*: monocytes, CD11c+ cells, and B cells (Fig. 4A). Of these, CD11c+ cells are the most abundant in the lungs during cryptococcal infection (Fig. 4A). Consequently, we interrupted the specific interaction between CD11c+ cells and Th cells by generating mice with conditional deletion of MHCII in cells that express CD11c (CD11c-cre MHCII fl/fl) (Fig. 4B). Unlike NOD/SCID/Rag mice that fail to generate mature Th cells, naïve CD11c-cre MHCII fl/fl mice produced an equivalent number of Th cells as naïve wildtype mice (Fig. 4C), showing the peripheral Th cell compartment remained intact in CD11c-cre MHII fl/fl mice. Thus, conditional deletion of MHCII on CD11c+ dendritic cells allowed specific disruption of the interaction between the dendritic cells and the Th cells in the periphery. Pulmonary Th cell expansion during cryptococcal infection was completely abolished in CD11c-cre MHCII fl/fl mice (Fig. 4C-D). Consequently, MHCII-bearing CD11c+ cells prime antigen-specific Th cells in the lungs of mice infected with *C. neoformans*.

CD11c+ cells are a heterogeneous group of macrophages and several dendritic cells (DC) subsets in the lungs [24]. Therefore, we sought to discern the specific lineage of the CD11c+ antigen presenting cell that is responsible for pulmonary Th2 cell induction using an unbiased forward genetic screen of mouse lines genetically deficient in various CD11c+ subsets or their ability to interact with Th cells via MHCII (S5A Fig.). Lysozyme M (LysM)-cre MHCII fl/fl (macrophages and granulocytes [25]), BATF3−/− (CD103+ conventional dendritic cells [5]), and CCR2−/− (monocytes and monocyte-derived dendritic cells [26]) mice generated robust antigen-specific Th2 responses during cryptococcal infection (S5–S6 Fig.). Only mice deficient in CD11b+ conventional dendritic cells, abrogated using CD11c-cre IRF4 fl/fl mice, experienced blunted Th2 cell accumulation with cryptococcal infection (Fig. 4E-H). Therefore, our exhaustive search revealed lung-resident CDllc+ CDllb+ IRF4-dependent conventional DC (referred to as CD11b+ conventional DC) are uniquely required for Th2 cell induction in response to pulmonary cryptococcal infection.

### Fungal chitin correlates with Th2 cell induction and subsequent disease

Existing evidence and our data show CD11b+ conventional dendritic cells are capable of inducing both Th17 and Th2 cell responses to pulmonary fungal infection [27]; therefore, these DC are not inherently programmed to specify a single Th cell lineage. Determination of Th2 cell fate by these lung resident DC must require higher order detection of specific features of the fungal infection. Many chitin-containing pathogens, as well as asthma/allergy models using purified chitin, evoke type-2 immunity [9,16,28]. Consequently, the striking Th2 cell response to pulmonary fungal infection prompted us to explore the role of chitin as a Th2 cell adjuvant.

Maintaining chitin homeostasis is critical for cell wall integrity and microbe vigor. Chitin synthases that regulate cell wall chitin deposition are often essential for fungal viability or are part of a redundant pathway [29]. As a result, studies that attempt to correlate loss-of-function mutations in chitin synthesis genes with modulation of the host response and attenuation of virulence would be challenging to interpret. Whether loss of Th2 cells and alterations in disease were due to a decrease in chitin, a loss of cryptococcal fitness/growth, or unmasking of other antigens due to modifications of the fungal cell wall or capsule would not be easily distinguishable [30].

**Fig 5:**
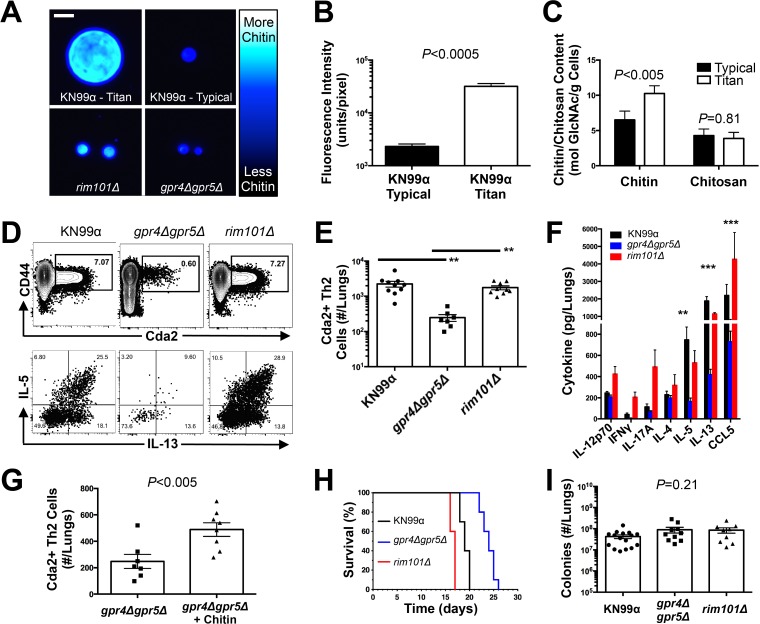
*C*. *neoformans* Chitin Correlates with Type-2 Helper T Cell Response and Subsequent Disease. (**A&B**) Cryptococcal cells isolated from lungs at 14 days post-infection and stained with Calcofluor White. Cells were either imaged with epifluorescence microscopy (**A**) or analyzed by flow cytometry with fluorescence intensity calculated per pixel to determine chitin density within the cell wall (**B**). (**C**) Total chitin and chitosan content normalized to dry weight of cryptococccal cells. (**D**) Flow cytometry plots of CD4+, Foxp3-, CD44+ Cda2+ Th cells expressing Th2 cytokines, IL-5 & IL-13, at 14 days post-infection and (**E**) the quantification of these plots. (**F**) Cytokines from lung homogenates of mice 14 days post-infection. (**G**) IL-5+ IL-13+ antigen-specific Th2 cells from lungs of mice 14 days post-infection without and with intranasal chitin particle treatment. (**H**) *P*-value represents log-rank test comparing each survival curve with 10 mice per group to: *gpr4Δgpr5Δ* vs. KN99α, P<0.0005; *gpr4Δgpr5Δ* vs. *rim101Δ, P*<0.0005; KN99α vs. *rim101Δ, P*<0.0005. (**I**) Fungal burden in the lungs at 14 days post-infection. Data are presented as the mean +/- standard error with at least 2 independent experiments per group. ** = *P* < 0.005, *** = *P* < 0.0005 by Mann-Whitney *U* or Kruskal Wallis ANOVA. Cda = chitin deacetylase, CCL = chemokine ligand, GlcNAc = *N*-acetylglucosamine, IFN = interferon, IL = interleukin.

In lieu of testing the requirement of chitin in Th2 cell induction, we exploited a natural property of *C. neoformans* to determine if increased fungal chitin was sufficient to expand Th2 cell formation. Approximately 20% of wildtype cryptococcal cells (KN99α) recovered from the lungs of infected mice increase in diameter from <10 μm to 15–100 μm [31–33]. Previous studies have shown these enlarged cells, known as titan cells, exhibit increased thickness of the fungal cell wall [32]. Using the fluorescent dye calcofluor white to measure chitin content in individual cells by epifluorescence microscopy (Fig. 5A-B), or at the population level with flow cytometry (S7A Fig.), we found the large *C. neoformans* titan cells contained more chitin, at a higher density, than the typical sized cells. *C. neoformans* produces several enzymes that deacetylate chitin to form chitosan [18,30]. Biochemical analyses additionally revealed that the amount of chitosan produced by titan cells and typical size cells did not differ, whereas chitin was significantly more abundant in the titan cells (Fig. 5C). Therefore, enhanced chitin content accompanied cell size increases during formation of cryptococcal titan cells.

To control for the relative effects of cell size and chitin content on Th2-mediated disease, we utilized several mutants of *C. neoformans*. A strain with targeted deletions in G protein-coupled receptor (*gpr*) 4 & *gpr*5 produces >95% typical sized cells *in vivo* [34], and these cells retain normal amounts of chitin (Fig. 5A). Deletion of the transcription factor Rim101 (*rim101Δ*) abolishes titan cell production [34,35], yet the typical sized cells have increased expression of chitin synthesis genes and elevated chitin content [36,37] (Fig. 5A). Using these mutants, we were able to dissociate cryptococcal cell size from cell wall chitin as well as manipulate the total amount of chitin present during infection (Table 2).

**Table 2:** Differences in total chitin due to cell morphology.

Strain	Proportion Titan Cells^a^	Chitin Per Cell (GMFI)^b^	Titan Cell Chitin (GMFI)	Typical Cell Chitin(GMFI)	Relative Total Chitin^c^
**KN99α**	20.4%	47609 (Titan) 3060 (Typical)	9712	2436	4.0
***gpr4Δgpr5Δ***	5.3%	43590 (Titan) 3102 (Typical)	2310	2938	1.7
***rim101Δ***	0.5%	45708 (Titan) 13112 (Typical)	2235	12456	4.8

^a^ Proportion of cryptococcal titan cells in the lungs, as reported by Okagaki et al. 2011[34].

^b^ Mean fluorescence intesity of calcofluor white in size-fractionated cryptococcal cells analyzed by flow cytometry. GMFI = geometric mean fluorescence intensity

^c^ Estimated total chitin calculated uising the folowing equation: Relative Total Chitin = [(Proportion Titan Cells × Titan GMFI) + (Proportion Typical Cells × Typical GMFI)]/KN99α Typical Cell GMFI

We examined the impact of alterations in chitin content in *C. neoformans* on Th2 cell accumulation in the lungs. Antigen-specific Th cell priming and Th2 cell differentiation were reduced in mice infected with the low chitin *gpr4Δgpr5Δ* strain compared to infections with both the high chitin KN99α (due to titan cell production) and *rim101Δ* strains (Fig. 5D-E). The Cda2-independent polyclonal Th2 cell response to *gpr4Δgpr5Δ* infection was also significantly lower than the responses to KN99α and *rim101Δ* infections (S7B Fig.), indicating the defect in antigen-specific Th2 cell accumulation to *gpr4Δgpr5Δ* infection was not due to differential expression of *cda2* between the strains. Finally, the secreted Th2 cytokines IL-5, IL-13, and CCL5 present in lung homogenates from mice infected with *gpr4Δgpr5Δ* were significantly reduced compared to KN99α and *rim101Δ* infections (Fig. 5F). Of note, secreted Th1 and Th17 cytokines, interferon-γ and IL-17A, did not concomitantly increase in response to *gpr4Δgpr5Δ* infection (Fig. 5F). Thus, Th cells not receiving strongly polarizing Th2 signals in the *gpr4Δgpr5Δ* infection failed to acquire an alternate Th1 or Th17 cell differentiation fate. Since KN99α and *rim101Δ* strains contribute more total chitin to the infection than the *gpr4Δgpr5Δ* strain (Table 2, S7A Fig.), these data demonstrate that chitin abundance and not cell size positively correlated with Th2 cell response intensity.

Uncontrolled factors affected by the *gpr4Δgpr5Δ* or *rim101Δ* mutations could correlate with chitin levels and independently contribute to Th2 cell accumulation. To directly test if purified chitin can increase Th2 cell numbers, mice were infected with *gpr4Δgpr5Δ*, and <10 μm chitin particles were co-administered into the lungs. Chitin treatments partially rescued the attenuated Th2 cell response (Fig. 5G). Importantly, this effect was observed in the clonal population of antigen-specific cells, and therefore, chitin increased the potency of Th2 cell induction during *C. neoformans* infection.

We examined the correlation between chitin, Th2 cell accumulation, and disease severity in our experimental model of cryptococcal infection. Infections with the high chitin strains, KN99α and *rim101Δ*, significantly hastened the time to death relative to mice infected with *gpr4Δgpr5Δ* (Fig. 5H). Interestingly, all strains had equivalent pulmonary colony forming units at 14-days post-infection, indicating the differences in disease were not simply due to a failure to control the infection (Fig. 5I), but rather paralleled the total amount of chitin present during infection (Table 2). In summary, both chitin production by *C. neoformans* and Th2 cell accumulation directly correlated with exacerbation of lethal fungal disease.

### Chitotriosidase deficiency confers resistance to Th2-mediated fungal disease

**Fig 6:**
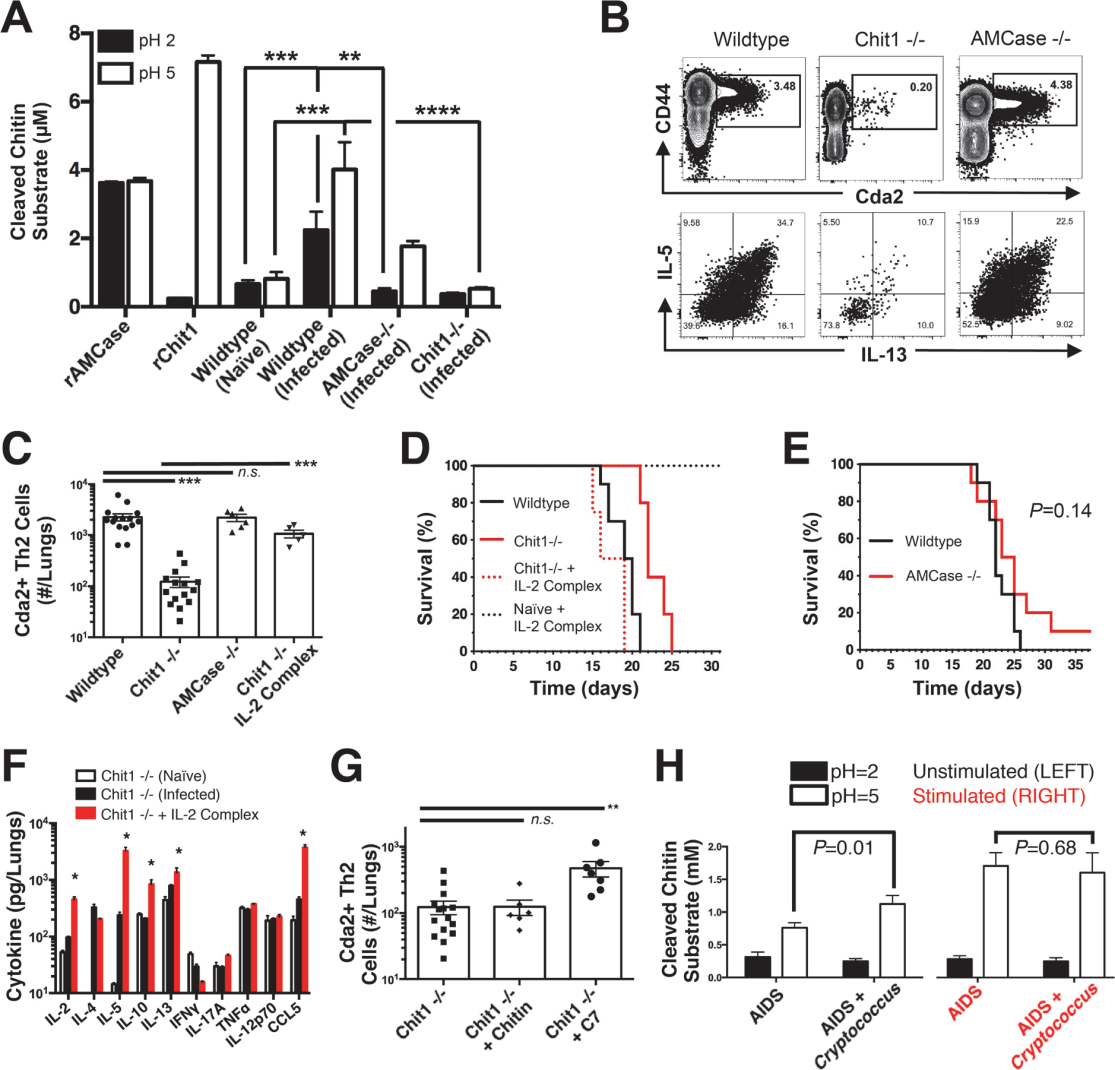
Chitotriosidase Promotes Chitin Recognition and Th2 Cell-mediated Disease. (**A**) Chitinase enzyme activity of recombinant enzymes or lung homogenates from 14 days post-infected or naïve mice. (**B**) Flow cytometry plots of CD4+, Foxp3-, CD44+ Cda2+ Th cells expressing Th2 cytokines, IL-5 & IL-13, at 14 days post-infection and (**C**) the quantification of these plots. (**D&E**) Survival of mutant or wildtype mice infected with KN99α either with or without IL-2 complex treatment. Logrank test comparing each survival curve relative with 10 mice for each group to: Chit1 −/− vs. wildtype, *P*<0.005; Chit1 −/− vs. Chit1 −/− + IL-2 complex, *P*<0.0005; Chit1 −/− + IL-2 complex vs. wildtype, *P* = 0.07. (**F**) Cytokines from lung homogenates of naïve or wildtype and Chit1−/− mice 14 days post-infection with KN99α or age-matched, naïve Chit1−/− mice. (**G**) IL-5+ IL-13+ antigen-specific Th2 cells from lungs of Chit1−/− mice 14 days post-infection and mice treated with intranasal chitin particles or chitin heptamer fragments (C7). (**H**) Chitinase enzyme activity of human plasma without (LEFT) or with (RIGHT) cryptococcal lysate antigen stimulation. Data are presented as the mean +/- standard error with at least 2 independent experiments per group. * = *P* < 0.05, ** = *P* < 0.005, *** = *P* < 0.0005 by Mann-Whitney *U* or Kruskal Wallis ANOVA. AMCase = Acidic Mammalian Chitinase, C7 = chitin heptamer, CCL = chemokine ligand, Cda = chitin deacetylase, Chit1 = Chitotriosidase, IFN = interferon, IL = interleukin, TNF = tumor necrosis factor.

We next investigated host intrinsic factors that could influence detrimental Th2 cell responses to chitin—specifically the mammalian chitinases, AMCase and Chit1. pH-sensitive differences in enzyme activity allow for an assessment of the contribution of each enzyme to chitin cleavage. Consistent with published reports, recombinant AMCase cleaved 4-methlylumbelliferone chitotriose across a broad pH range, whereas recombinant Chit1 was only active at less acidic pH (Fig. 6A) [38]. Chitinase activity in lung homogenates from infected mice was significantly elevated compared with uninfected animals at pH 2 and pH 5 (Fig. 6A). Furthermore, lung homogenates from infected mice genetically deficient in Chit1 or AMCase both showed decreased cleavage of the fluorescent chitin substrate (Fig. 6A). These data indicate both chitinase enzymes are active during pulmonary fungal infection, and genetic abolishment of these enzymes can be used to understand the effect of chitin degradation on Th2-mediated fungal pathogenesis.

To test the hypothesis that Th2 cell-associated disease depends on chitinases, we infected wildtype, Chit1−/−, and AMCase−/− mice with *C. neoformans* and quantified the Th2 cell response. Despite no differences in pulmonary fungal burden at 14-days post-infection (S8A Fig.), Th2 cells were 10-fold less abundant in the lungs of infected Chit1−/− mice compared with wildtype controls (Fig. 6B-C), and this trend was consistent with all the strains of *C. neoformans* tested (S8B Fig.). Conversely, AMCase deficiency did not impact Th2 cell quantities after cryptococcal infection (Fig. 6B-C). Furthermore, Chit1 deficiency and not AMCase deficiency also significantly extended the survival of mice infected with *C. neoformans* relative to age matched, wildtype animals (Fig. 6D-E). The loss of Th2 cell accumulation with Chit1 deficiency was responsible for attenuation of disease, because the use of IL-2 complexes to boost Th2 cell numbers (Fig. 6C) and associated cytokines (Fig. 6F) also hastened lethal disease in Chit1−/− mice compared with infection-matched, untreated Chit1−/− controls (Fig. 6D). Similar to our previous studies using the IL-2 complex, Th1 cytokine production in the Chit1−/− mice was only minimally affected by the IL-2 complex treatment (Fig. 6F). Thus, the presence of Chit1, and not AMCase, positively influences Th2 induction and subsequent disease.

### Digestion of chitin via chitotriosidase promotes Th2 cell accumulation

Chitin receptors in plants bind chitin oligomers [39], and chitin polymer size also influences the mammalian immune response to chitin [40,41]. Since appropriately sized chitin fragments could result from chitin digestion by Chit1, we used heterogeneous-length chitin and highly purified chitin heptamers to understand the effect of Chit1-associated degradation of chitin on Th2 cell accumulation. Although treatment with heterogeneous-length chitin augmented Th2 cell induction in wildtype animals infected with *gpr4Δgpr5Δ* cells (Fig. 5F), it did not increase Th2 cell numbers in Chit1−/− mice (Fig. 6G). Conversely, inoculation with mass equivalent amounts of chitin heptamers boosted the Th2 cell response in *C. neoformans*-infected animals with Chit1 deficiency (Fig. 6G), revealing the requirement for Chit1 in chitin polymer recognition can be bypassed by providing exogenous chitin fragments. Therefore, our data demonstrate a role for Chit1 in the chitin cleavage pathway that leads to Th2 cell accumulation.

We next examined how chitin fragments influence the upstream pathway of Th2 cell induction. To test the hypothesis that DC interact directly with chitin fragments, we cultured primary pulmonary leukocytes from infected mice in the presence of R-phycoerithrin-fluorophore conjugated chitin heptamers (RPE-GN7) or unbound REP-streptavidin (RPE-SA). While RPE-GN7 labeled a subset of B cells that have been shown to bind chitin [9], RPE-GN7 did not adhere to conventional CD11b+ DC (S9A Fig.). Thus, we did not detect direct binding of chitin heptamers to the conventional DC. Furthermore, conventional CD11b+ DC stimulated *ex vivo* with PMA + ionomycin produced an important Th2 cell differentiation cytokine, IL-4, yet this DC subset did not express IL-4 upon stimulation with chitin heptamers (GN7) (S9B Fig.). Combined, these data suggest the CD11b+ conventional dendritic cells may not sense chitin levels directly.

Pulmonary epithelial cells respond to chitin [42] and secrete several Th2-inducing alarmins, thymic-stromal lymphopoietin (TSLP), IL-25, and IL-33 [28,43,44]. As a result, alarmin receptors on DC could potentially mediate the indirect recognition of chitin, leading to Th2 cell polarization during pulmonary fungal infection. We found CD11b+ conventional DC from the lungs of fungal infected mice expressed high levels of TSLP receptor, but not receptors for IL-25 or IL-33 (S9C–S9D Fig.), indicating this DC subset is capable of sensing TSLP generated by epithelial cells. Taken together, our data suggest Th2 cell induction by conventional CD11b+ DC appears to involve an indirect recognition of chitin oligomers.

### Chitotriosidase activity correlates with cryptococcal infection in humans

The importance of Chit1 in promoting Th2 cell-mediated disease in an experimental model of cryptococcosis prompted us to investigate the relevance of chitotriosidase activity in human fungal disease. Blood samples were collected from human donors: 46 Ugandan patients presenting at the hospital for the first time with AIDS and 38 similar AIDS patients experiencing acute cryptococcal disease (S1 Table). We analyzed chitotriosidase and AMCase enzymatic activity in plasma from each group as described for the mouse lung homogenates. Chitin substrate cleavage at pH = 5 was significantly elevated in plasma from AIDS patients with cryptococcal infection when compared to AIDS patients without cryptococcal infection (Fig. 6H). Comparatively low levels of enzymatic activity were detected at pH = 2 for each group, indicating that chitotriosidase and not acidic mammalian chitinase is the predominate chitinase produced by humans with cryptococcal infection (Fig. 6H).

To determine if the difference in chitotriosidase activity was due to an inherent propensity or deficiency in chitotriosidase expression, we used cryptococcal lysate antigens to stimulate chitinase production in whole blood samples from the human donors. Stimulation of whole blood from all patients induced robust chitotriosidase activity relative to unstimulated samples, and chitinase activity did not differ between the groups (Fig. 6H), indicating all human donors had equivalent capacity to produce chitotriosidase. Chitinase activity was not detectable in pure cryptococcal culture supernatants or cryptococcal lysate antigens (S10 Fig.), and as a result, the chitinase activity detected in the assays with human samples was not due to *Cryptococcus*-derived chitinases. Taken together, we conclude that fungal antigen induces chitotriosidase activity in humans experiencing cryptococcosis.

## Discussion

An association between pulmonary fungal exposure and allergic Th2 inflammation is well established [1,3]. Fungal proteases [45] and fungal chitin [46] impact the innate immune responses underlying allergic inflammation, but elements of Th2 cell induction are enigmatic. Using an experimental model of pulmonary cryptococcosis, we demonstrated that inhalation of *C. neoformans* establishes a robust pulmonary infection, and the potent antigen-specific Th2 cell accumulation required lung resident CD11b+ conventional DC. Since these CD11b+ conventional DC can stimulate Th2 or Th17 cell differentiation in response to *C. neoformans* and *Aspergillus fumigatus* exposure respectively [27], these DC must interpret specific features of the infection to direct Th2 cell fate. To this end, we found cryptococcal chitin and exogenous administration of chitin particles correlated with increased Th2 cell accumulation. We further showed that chitotriosidase activity was highest in mice and humans infected with *C. neoformans*, and Chit1 was necessary for efficient Th2 cell induction and disease in our murine model of cryptococcosis. Taken together, these data indicate the host response to fungal chitin is an important factor that enhances Th2 cell production during pulmonary fungal infection.

**Fig 7:**
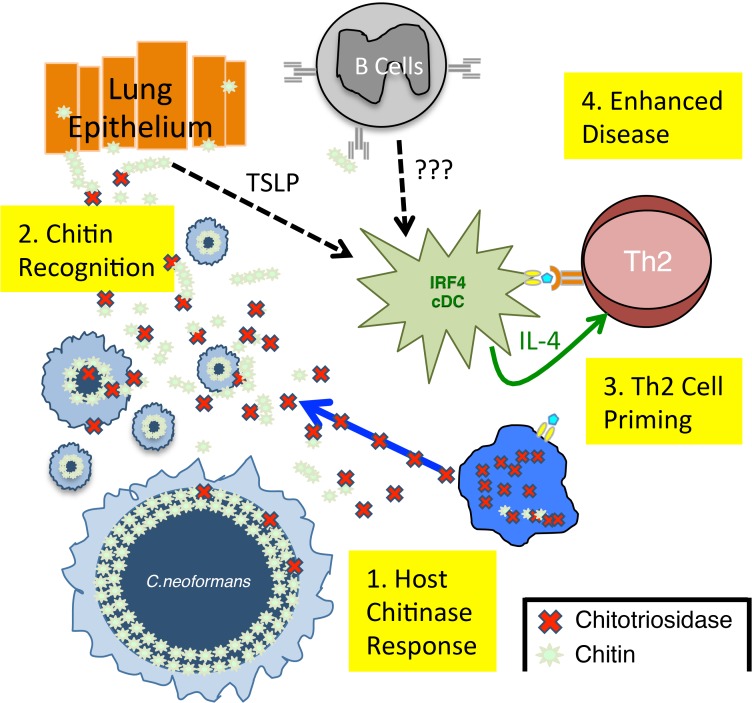
Model of Th2 Cell-mediated Disease during Pulmonary Fungal Infection. **1**) Chitotriosidase is released by the host and degrades fungal chitin to generate small chitin fragments, such as chitin heptamers. **2**) Chitin recognition occurs by an unknown mechanism that could involve either epithelial cell production of alarmins, such as thymic stromal lymphopoietin (TLSP) [9] or antibodies that bind chitin [9]. **3**) These chitin-based signals are recognized by lung-resident CD11b+ conventional dendritic cells that are capable of producing IL-4, an essential Th2 cell differentiation factor. **4**) The adaptive Th2 cell response results in enhanced disease in the absence of significant changes in pulmonary fungal burden.

Our findings narrow an important gap in the mechanism of pattern recognition of fungal chitin in the lungs (Fig. 7). We have shown Chit1 functions as a “gatekeeper” in making chitin fragments available to host surveillance, thereby promoting Th2 cell accumulation and disease. Additionally, the use of pharmaceutical grade chitin heptamers to augment Th2 cell responses establishes a new minimum component for chitin recognition in vertebrates. While we did not detect direct interactions between the CD11b+ conventional DC and labeled chitin *ex vivo*, other systems have shown chitin binding to the mannose receptor and subsequent recognition by TLR9 and/or NOD2 [47]. Alternatively, pulmonary allergy models demonstrate that lung epithelial cells recognize chitin fragments and produce the necessary alarmins for Th2 cell induction: TSLP, IL-25, and IL-33 [43,44,48]. In our model, CD11b+ conventional DC express high levels of TSLPR. Finally, natural IgM has been shown to bind fungal carbohydrates, including chitin, and facilitate the interaction of DC and fungal carbohydrates [9]. Signals, such as alarmins or antibody-chitin complexes, could be received by CD11b+ conventional DC to direct Th2 cell differentiation via IL-4 or another novel pathway.

A major impediment in understanding Th cell responses to pulmonary fungal infection has been the lack of reagents to detect antigen-specific Th cells. Antigen-specific reagents are particularly important when examining Th2 responses, because Th2 cells can be induced by the wounding that occurs during infection. Our ability to track endogenously derived, antigen-specific Th cells with pMHCII tetramers [49] allowed us to present for the first time that Th2 cells produced in response to fungal pathogens are not part of a generalized wound healing response but are fungal-antigen specific. Unlike T cell receptor transgenic approaches, pMHCII tetramers permitted us to monitor the population of infection-specific Th cells in the polyclonal repertoire, while maintaining physiologic precursor frequency and clonal expansion. Thus, we are able to examine the Th cell response during the natural course of infection and keep all other variables constant. The availability of these pMHCII tetramers will undoubtedly empower cryptococcal researchers and accelerate the field of fungal immunology.

The use of IL-2 complexes allowed us to conveniently and reliably augment the Th2 cell response to further understand unappreciated elements underlying Th2-mediated disease. This strategy is amenable to any host or microbial genetic model, which facilitates direct comparisons. Also, this gain-of-function approach permitted us to test the sufficiency of Th2 cells to exacerbate disease. These data, combined with loss-of-function studies by other groups [50,51], alter the longstanding paradigm that susceptibility to lethal fungal disease is traditionally viewed as a breakdown in protective immunity. This paradigm is supported by the higher prevalence of invasive fungal disease in immunosuppressed individuals, including people living with HIV/AIDS, cancer patients undergoing chemotherapy, and solid organ transplant recipients. However, we propose that in addition to the lack of a protective response, an independent development of a harmful Th2 cell response further exacerbates disease. This is particularly important in the the case of human cryptococcocosis were a compromised immune system not only lacks sufficient quantities of lymphocytes to resolve the fungal infection, but the residual Th cell repertoire is plastic and detrimentally influenced by the microbe [52–55].

A subset of innate lymphoid cells (ILC) produce the Th2 cytokines, IL-5 and IL-13 [56], and these so-called ILC2 have been shown to contribute to allergic airway disease [4]. While IL-2 complex treatment dramatically increases Th2 cell accumulation and enhances pulmonary disease, ILC numbers are not affected by IL-2 complex treatment in our model. Likewise, CD25+ ILC2 exist in the lungs under homeostatic conditions [57], yet uninfected mice exhibit no ill effects of IL-2 complex treatment. However, the developmental relationship of ILC2 to lymphocytes, combined with the lack of lineage markers expressed by ILC, make it challenging to separate the relative effects of Th2 cells and ILC2 in driving immunopathology. Thus, our conclusion that Th2 cells are vital mediators of disease does not categorically exclude the participation of ILC2 in this process.

CD11b+ conventional DC are an ontologically distinct mononuclear phagocyte subset that require IRF4 for maturation [5]. Although it has been suggested that this subset is also functionally unique in programing specific Th cell differentiation, building evidence seems to indicate a plastic role of these cells in Th cell induction. CD11b+ conventional IRF4-dependent DC have been shown to coordinate Th2 cell priming following protease inoculation and worm infection in the skin [58], as well as house dust mite extract installation in the lungs [59]. However, several research groups have found the same DC subset, using identical host genetic systems, can control Th17 cell differentiation in the gut under homeostatic conditions [60] and the lungs after fungal infection [27]. While these observations infer that CD11b+ conventional DC are plastic, these cells may have different functions in the skin and gut compared to the lungs that could explain their role in priming Th17 and Th2 cell responses. Our findings showing Th2 priming by *C. neoformans*, combined with work by Schlitzer et al. showing IRF4-dependent DC are capable of priming Th17 cells in response to *A. fumigatus* to pulmonary fungal infection, highlight a fatal flaw in the notion that DC subsets are inherently specialized to control Th cell lineage fate. As a result, we explored an alternative hypothesis that higher order signals (e.g. chitin recognition) are required for pulmonary CD11b+ conventional DC to promote Th2 cell priming to fungal infection.

Gene deletions in microbes can cause pleiotropic phenotypes, as seen in the *rim101Δ* strain (35) that can complicate interpretation of the effects of these mutations on host responses. However, the data obtained from the mutants utilized in this study support the conclusion that fungal chitin promotes Th2-mediated disease for several reasons. First, equivalent fungal burden in the lungs at 14 days post-infection indicates these mutations do not confer an inherent survival advantage or disadvantage for the fungus, and any effect the mutants have on leukocyte accumulation or disease is not simply driven by antigen load. Second, Rim101 transcriptionally controls many elements associated with cryptococcal virulence, including pH responses, encapsulation, cell enlargement, and iron sequestration [61]. While individual mutations in each of these pathways should result in a loss of virulence, the *rim101Δ* mutants paradoxically exhibit equivalent or accelerated disease [36]. Altered expression of cell wall synthesis genes combined with unmasking of the cell wall has been posited [36,37], and in particular, our data implicate cell wall chitin in enhancing fungal pathogenesis associated with *rim101* deficiency. More importantly, we view *rim101Δ* as an essential control for the effect cryptococcal cell size has on Th2 cell formation more than an independent test of the hypothesis that fungal chitin drives Th2 cell priming. Thus, we needed a strain of *C. neoformans* that produces mostly small cells but retained elevated chitin density to offset the loss of elevated chitin density with titan cell deficiency. At a minimum, these data prove that large cryptococcal cell size is not driving robust Th2 cell accumulation. Finally, due to the structural similarities, as well as the interdependent synthetic pathways, it is extremely challenging to decouple how chitin and chitosan separately impact a complex biological system. Although we cannot rule out immunomodulatory effects of chitosan in our experiments with *C. neoformans* mutants, we have confirmed chitin alone, when provided as an adjuvant, is sufficient to augment Th2 cell accumulation. Interpretation of the *Cryptococcus* chitin mutant data in the context of our additional data regarding host recognition of exogenous chitin builds a compelling argument that fungal chitin promotes detrimental Th2 cell induction.

Acidic mammalian chitinase has been previously implicated in innate type-2 responses [62]. However, a head-to-head comparison of the effect of both mammalian chitinases, chitotriosidase and acidic mammalian chitinase, has never been performed in any model, much less in the context of Th2 cell responses to fungal infection. Surprisingly, chitotriosidase, and not acidic mammalian chitinase, influenced Th2 cell accumulation and disease during pulmonary fungal infection. While no direct explanation exists, the different patterns of expression of AMCase and Chit1 could explain our results. AMCase is produced by a number of cells including several leukocyte subsets and pulmonary epithelial cells, whereas Chit1 expression is restricted to mononuclear phagocytes, including DC [38,63]. Since these DC are the main coordinators of antigen presentation to CD4+ T cells, the close proximity of chitin degradation and recognition likely allows the DC to efficiently influence the fate of CD4+ T cells during pulmonary fungal infection. Furthermore, the varying pH-dependent enzymatic activity of Chit1 and AMCase suggest these enzymes may function in disparate anatomical and subcellular compartments that may bias their participation in response to pulmonary fungal infection.

Considering Th2 cell responses likely evolved to resist parasitic infection [10], the asymmetric global distribution of *CHIT1* alleles [64] combined with the data presented herein offers a unique perspective on why individuals from tropical regions with endemic parasites tend to experience frequent and severe mycosis [65–69]. Ethnic groups historically residing in regions with highly prevalent parasites like *Strongyloides* tend to maintain functional *CHIT1*, whereas populations from more temperate climates with lower endemic parasitic burdens frequently possess enzymatically inactive *CHIT1* alleles [64]. Perhaps continuous fungal exposure in the absence of parasitic encounters provides sufficient negative selection pressure to eliminate functional *CHIT1* alleles from these populations (e.g. Europe). Conversely, ethnic groups historically residing in tropical areas (e.g. Africa) that maintain functional *CHIT1* alleles may have enhanced protection against common parasitic exposures, while these same individuals experience exacerbation of Th2-mediated fungal disease [67–69].

In conclusion, we elucidated a novel mechanism of Th2 cell induction during fungal infection. CD11b+ DC, and importantly recognition of chitin via Chit1, drive the generation of deleterious Th2 cells responding to pulmonary fungal infection. Next generation anti-fungal treatments should not only block fungal growth, but should also target the host immune response. A recent trial used IFNγ in combination with traditional anti-fungal therapy to promote beneficial immune responses. This treatment improved cryptococcal clearance, yet it had no significant impact on patient survival [70]. Our study suggests treatments that additionally aim to suppress the pathologic Th2 response, perhaps through chitotriosidase inhibition, may be necessary to improve clinical outcomes. Ultimately, the coordinated efforts of microbiologists, immunologists and infectious disease physicians will enable personalized medicine approaches that effectively combat lethal fungal infections by inhibiting fungal growth, promoting beneficial host responses, and dampening pathologic inflammation.

## Materials and Methods

### Ethics statement

This study was approved by the institutional review boards of the University of Minnesota, Makerere University, and the Uganda National Council of Science and Technology. Written informed consent was obtained from all human participants prior to inclusion in the sutdy, and all data were de-identified [71]. All animal experiments were done in concordance with the Animal Welfare Act, U.S. federal law, and NIH guidelines. Mice were handled in accordance with guidelines defined by the University of Minnesota Institutional Animal Care and Use Committee (IACUC) protocol numbers 1010A91133 and 1207A17286 and University of Massachuesets IACUC protocol number A-1802.

### Mice

All mice used in this study were derived from a C57BL/6 background. C57BL/6J, LysM-cre (B6.129P2-*Lyz2* ^*tm1(cre)Ifo*^/J), CD11c-cre (B6.Cg-Tg (Itgax-cre) 1-1Reiz/J), MHCII *loxP* (B6.129X1-*H2-Ab1* ^*tm1Koni*^/J), Batf3 −/− (B6.129S(C)-*Batf3* ^*tm1Kmm*/J^), CCR2 −/− (B6.129(Cg)-*Ccr2* ^*tm2*.*1Ifc*/J^), B6.129(Cg)-*Foxp3* ^*tm3(DTR/GFP)Ayr*^/J) mice were purchased from Jackson Laboratories (Bar Harbor, ME) and Flt3L−/− (C57BL/6-_*flt3L*_ *tm1Imx*) were purchased from Taconic (Hudson, NY). Crosses were performed when necessary to generate the mouse strains used in this study, as indicated in S2 Table. Chit1 −/− [72] mice were infected and processed in the laboratory of Kirsten Nielsen. AMCase−/− [73] mice were infected and processed in the laboratory of Stuart Levitz per MTA stipulations. All mice were housed in specific pathogen–free conditions.

### *Cryptococcus*

*Cryptococcus neoformans* var. *grubii* strains were streaked on yeast peptone dextrose (YPD) agar plates and incubated for 2 days at 30°C. YPD broth was inoculated with colonies from the aforementioned plate and incubated for 16 hours at 30°C with gentle agitation. The inoculum was prepared by pelleting the culture, washing 3 times with phosphate buffered saline (PBS), and resuspending in PBS at a concentration of 2x10^6^ cells/mL. All strains used in this study were on a KN99α genetic background, and the complemented strains have wildtype phenotypes [18,34,35].

### Infection

A well established intranasal pulmonary aspiration model of cryptococcosis was used for this study [74]. 6–8 week old, sex-matched mice were anesthetized with pentobarbitol or isoflurane. 5x10^4^ cryptococcal cells in 25 μL of PBS was placed on the nares of each mouse, and the mice aspirated the inoculum into the lower respiratory tract. Finally, the mice were suspended by their incisors for 5 minutes and subsequently placed upright in their cage until regaining consciousness. For survival studies, ten mice per group were infected as described above. Animals were monitored for morbidity and sacrificed when endpoint criteria were reached. Endpoint criteria were defined as 20% total body weight loss, loss of 2 grams of weight in 2 days, or symptoms of neurological disease.

### Tetramer production

Nine amino acid peptides from Cda2 were selected using a MHCII loading algorithm [75]. pMHCII tetramers were produced as previously described [76]. In short, biotinylated pMHCII monomers were expressed in *Drosophila melanogaster* S2 cells and isolated from culture supernatants by affinity chromatography. Streptavidin-Phycoerythrin (Prozyme) was added to pMHCII monomers at a 4:1 molar ratio. Finally, tetramer formation was assessed by western blot analysis.

### Pulmonary leukocyte preparation

Lung leukocytes were isolated as previously described [77]. Briefly, lungs were excised and minced to generate approximately 1 mm^3^ pieces. The lung mince was incubated in HBSS (Invitrogen, Grand Island, NY) + 1.3 mM EDTA solution for 30 min at 37°C with agitation, and then transferred to RPMI-1640 (Invitrogen) medium supplemented with 5% Fetal Bovine Serum (FBS) (Invitrogen) and 150 U/ml type I collagenase (Invitrogen) and incubated for 1 h at 37°C with agitation. The cells were passed through a 70 μm filter, pelleted, and resuspended in 44% Percoll-RPMI medium (GE Life Sciences, Pittsburgh, PA). A Percoll density gradient was created (44% top, 67% bottom), and the samples were centrifuged for 20 min at 650 x *g*. The leukocytes at the interface were removed, washed 2 times with RPMI medium, and resuspended in PBS + FBS at a concentration of 10^7^ cells/ml. CD4+ T cells were enriched using a Dynabeads CD4+ T Cell Negative Isolation Kit (Life Technologies, Grand Island, NY) per manufacture’s instructions. After enrichment, ∼10^6^ cells were suspended in 200 μL of restimulation buffer (RPMI + 10% FBS + 1% Penicillin/Streptomycin + 5 μg Brefeldin A) with (stimulated) or without (unstimulated) 10 ng phorbol myristate acetate (PMA) and 50 ng ionomycin. After 6 hours, the cells were washed and immediately prepared for flow cytometry.

### IL-2 complexes

5 μg of murine IL-2 cytokine (Biolegend) and 25 μg of clone JES6-1A12 anti-IL-2 antibody (Bio X Cell, West Lebanon, NH) were added to 100 μL of PBS at room temperature. Each mouse received intraperitoneal injections of IL-2 complexes every other day beginning at 5 days post-infection.

### Flow cytometry

Samples were incubated for 5 minutes with CD16/32 antibody (Biolegend) to block the Fc receptor and prevent nonspecific antibody binding. 25nM Cda2-tetramer was added to the sample and incubated at 25°C for 1 hour in the dark. Samples were surface-stained at 4°C for 30 minutes with the following antibodies (see gating S1 Fig.): CD4 (RM4-5, BV605, Biolegend), CD8 (53-6.7, APC-eFluor 780, eBioscience, San Diego, CA), CD11b (M1/70, PE-Cy5, eBioscience), CD11c (N418, PE-Cy5, eBioscience), B220 (RA3-6B2, PE-Cy5, eBioscience), and CD44 (IM7, Alexa Fluor 700, Biolegend). The cells were then incubated in Foxp3 Transcription Factor Buffer (eBioscience) at 4°C for 30 minutes. The cells were labeled with antibodes against the following intracellular antigens: Foxp3 (FJK-16s, FITC, eBioscience), IL-5 (TRFK5, APC, Biolegend), IL-13 (eBio13A, eFluor 450, eBioscience), IL-17A (TC11-18H10.1, BV650, Biolegend), and IFNγ (XMG1.2, PE-Cy7, eBioscience). 1:200 antibody concentrations were used for surface staining, and 1:100 antibody concentrations were used for intracelluar staining. For data acquisition, events from the entire sample (500,000–1,000,000) were collected on a BD FACSCanto II flow cytometer (BD Biosciences, San Jose, CA), and the data were analyzed with FlowJo X (Tree Star Inc., Ashland, OR).

To account for cell loss during CD4+ T cell enrichment and mitogen restimulation, several calculations were performed. Total leukocyte numbers were determined by hemacytometer count after Percoll density gradient separation. 2.5% of the sample was stained with the following antibodies: Ly6G (RB6-8C5, APC-eFluor 780, eBioscience), Ly6C (HK1.4, eFluor 450, eBioscience), CD11b (M1/70, BV650, Biolegend), CD11c (N418, BV605, Biolegend), NK1.1 (PK136, AF700, Biolegend), CD3 (17A2, PE-Cy5, Biolegend), CD4 (RM4-5, FITC, Biolegend), CD19 (6D5, PE-Cy7, Biolegend), Sca1 (D7, APC, Biolegend), and Siglec F (E50-2440, PE, BD Biosciences). Cells were identified as the following (see gating S2 Fig.): Th cells = CD11b- CD3+ CD4+. Eosinophils = CD11b+ CD11c- Siglec F+. Innate lymphoid cells = lineage- Sca1+. Dendritic cells and macrophages = CD11b+ CD11c+ Siglec F-. B cells = Siglec F- CD11b- CD11c- CD19+. Natural killer cells = Siglec F- CD11c- NK1.1+. Neutrophils = CD11b+ CD11c- Ly6G+. Monocytes = CD11b+ Ly6C+ Siglec F-. CD8+ T cells = Siglec F- CD11b- CD3+ CD4-. The proportion of CD4+ T cells was determined by flow cytometry, and this percentage was multiplied by the total number of lung leukocytes to calculate the number of CD4+ T cells per pair of lungs. The number of CD4+ T cells in the unstimulated, CD4+ T cell enriched sample was calculated after flow cytometric analysis, as described in the previous paragraph. The number of CD4+ T cells recovered in the unstimulated, enriched sample was divided by the total number of CD4+ T cells to calculate the CD4+ T cell isolation efficiency. Cell death due to mitogen restimulation was calculated by dividing the number of CD4+ T cells recovered in the stimulated sample by the number of CD4+ T cells recovered in the unstimulated sample. The number of Cda2+ Th2 cells was determined by dividing the number of Cda2+ IL-5 and/or IL-13+ positive cells by the CD4+ enrichment and cell viability indices.

Dendritic cell subsets were determined using the following antibodies and gating strategy. CD3 (17A2, PE-Cy5, Biolegend), CD19 (6D5, PE-Cy5, Biolegend), Siglec F (E50-2440, APC, BD Biosciences), CD64 (X54-5/7.1, PE, Biolegend), MHCII (M5/114.15.2, AF700, Biolegend), CD11c (N418, BV605, Biolegend), CD11b (M1/70, BV650, Biolegend), CD103 (2E7, e450, eBioscience), FcεRI (MAR-1, PE-Cy7, Biolegend), TSLPR (PE, R&D Systems), IL-25R (9B10, PE, Biolegend), IL-33Rα (DIH9, PE, Biolegend), IL-4 (BVD6-24G2), PE, Biolegend), and GN7-PE [78]. See gating S5B Fig. Dump = CD3+, CD19+, Siglec F+. Monocyte-derived DC = Dump-, CD64+, CD11c+, MHCII+, CD11b+, CD103-, FcεRI+. CD103+ cDC = Dump-, CD64-, CD11c+, MHCII+, CD103+, CD11b-. CD11b+ cDC = Dump-, CD64-, CD11c+, MHCII+, CD11b+, CD103-. While CD103, CD11b, FcεRI, and CD64 surface markers allow convenient detection of each DC subset, it is unclear whether other developmentally and/or functionally unrelated cells can express similar markers. Consequently, genetic blockade in the developmental pathways of each DC subset were assumed to be completely penetrant, notwithstanding the persistence of cells expressing surrogate markers for each ontological subset.

### Lung cytokines

Lungs from mice 14-days post-infection were excised, snap frozen in liquid nitrogen, and homogenized in 3 mL of T-PER (Thermo Fisher Scientific) with Complete Protease Inhibitor Cocktail (Roche, Indianapolis, IN). The lung homogenate was pelleted, and the supernatant was collected and stored at 80°C until analysis. Samples were diluted 1:4 in assay buffer immediately before processing. Cytokines were quantified using Luminex technology according to manufacturer instructions (Bio-Rad, Hercules, CA).

### Lung histology

Lungs were removed from mice 14 days post-infection, perfused via the right ventricle with cold PBS, inflated with 10% formalin (Thermo Fisher Scientific, Rockford, IL), and placed in a container of 10% formalin. Tissues were dried with organic solvent, embedded in parafin, sectioned, and stained with hematoxylin and eosin, before images were captured.

### Fungal chitin/chitosan quantification

Cryptococcal cells were isolated from the lungs after enzymatic digestion and density gradient separation, as described above. Cells were fixed with 3.7% formaldehyde and stored at 4°C until analyzed. The cells were standardized to 1x10^6^ cells/mL and stained for 2 minutes at 25°C with 1 μg/mL of Calcofluor White (Sigma Aldrich, St. Louis, MO) [79]. Cells were washed and immediately processed with an epifluorescence microscope (Axio Imager M1, 40X/0.6 lens, Zeiss Filter Set 02, Axio Cam MRc5, Axiovision 4.8; Carl Zeiss, Inc., Munich, Germany). ImageJ software (NIH.gov) was used to calculate fluorescence intesity per pixel. For flow cytometry, large and typical sized cells were first gated by forward scatter properties to distinguish size. Chitin/chitosan content was then determined by 405 nm laser excitation and fluorescence detection at ∼450 nm.

Biochemical chitin/chitosan quantification was adapted from Banks et al. [30]. Purified titan cell (>15 μm) and typical sized cell (<15 μm) samples collected from infected mice were each divided into two aliquots: one treated with acetic anhydride to fully acetylate the chitin/chitosan polymer and the other was left untreated. 5 μl of purified *Streptomyces griseus* chitinase (5 mg/ml in PBS) was added to hydrolyze chitin to *N*-acetylglucosamine (GlcNAc) and samples were incubated for 3 days at 37°C. For colorimetric determination of GlcNAc, the Morgan-Elson method was adapted for microplate readers. Chitinase-treated samples were incubated with 0.27 M sodium borate (pH 9.0) and heated at 99.9°C for 10 minutes. Immediately upon cooling to room temperature, freshly diluted 10X DMAB solution (10 g p-dimethylaminobenzaldehyde in 12.5 ml concentrated HCl and 87.5 ml glacial acetic acid) was added, followed by incubation at 37°C for 20 minutes. Absorbance at 585 nm was recorded for each sample. Standard curves were prepared from stocks of 0.2 to 2.0 mM of GlcNAc (Sigma). The amount of GlcNAc was calculated as mol/g cells (dry weight). The acetylated samples contained chitin plus chitosan, and the untreated sample contained chitin. The difference between the two measurements estimated the amount of chitosan.

### Exogenous chitin

Chitin was prepared as previously published [42]. Chitin from shrimp shells (Sigma Aldrich) was pulverized with a mortar and pestle. 12.5N HCl was added, and the slurry was incubated at 40°C for 30 minutes. Chilled 10N NaOH was added until a neutral pH was attained. The sample was centrifuged at 2x10^4^ *g* for 5 minutes, the supernatant was decanted, and the sample was suspended in deionized water (dH_2_O). This step was repeated 3 times followed by a wash in ethanol. The sample was pelleted, suspended in dH_2_O, and filtered through a 10μm membrane (EMD Millipore, Billerica, MA). The solution containing <10 μm chitin was dried with a SpeedVac (Thermo Scientific, West Palm Beach, FL). The powder was weighed and resuspended in PBS to make a concentration of 50 mg/mL (i.e. 10X). Endotoxin was measured by Limulus amebocyte lysate assay (Associates of Cape Cod, East Falmouth, MA) and was found to be less than 0.03 EU/mL. Chitin heptamers were purchased from Carbosynth (Berkshire, UK). Mice were anesthetized and allowed to aspirate 125 μg of chitin suspended in 25 μL of PBS into the lungs at 0, 5, and 10 d.p.i. Pulmonary leukocytes from wildtype mice 14 days post-infection with KN99α were cultured and stimulated *ex vivo* for 5 hours with PMA + ionomycin (as previously described for Th cells) + 125 μg of chitin heptamers + golgi stop, or golgi stop alone (unstimulated) before processing by flow cytometry.

### Chitinase activity

CycLex Acidic Mammalian Chitinase (AMCase) and Chitotriosidase (Chit1) Fluorometric Assays (MBL International, Woburn, MA) were used to detect chitinase activity. In brief, each of the following were added to pH 2 and pH 5 buffers containing 4-Methylumbelliferyl Chitotriose: 25 ng of recombinant AMCase, 25 ng recombinant Chit1, 10 μL of mouse lung homogenate, 10 μL of human plasma, 10 μL of lysate antigen, or 10 μL of culture supernatant of KN99α grown in YPD. The samples were incubated at 37°C in a Synergy H1 Microplate Reader (Biotek, Winooski, VT) and 360 nm excitation/450 nm emission readings were obtained every 2 minutes. The relative fluorescent units (RFU) at 1 hour of incubation were compared to the RFU of serial dilutions of 4MU standard, and the molar concentration of cleaved chitin was calculated.

### Antigen stimulation of whole blood

The assay was performed as previously described [55]. Cell wall antigens were prepared from *Cryptococcus neoformans*, strain KN99α. The cells were flash frozen in liquid nitrogen, combined with glass beads, and vortexed vigorously for 2 hours at 4°C to disrupt the cells. The insoluble fraction (i.e., cell wall) was analyzed for protein concentrations (bicinchoninic acid protein assay; Thermo Fisher Scientific, Rockford, IL). Endotoxin levels in all antigen preparations were undetectable (<0.06 U/ml) by *Limulus* amebocyte lysate assay (Associates of Cape Cod, East Falmouth, MA). Whole-blood samples were obtained from AIDS patients at screening for the *Cryptococcus* Optimal Timing of Anti-retroviral Therapy Trial in Sub-Saharan Africa [71]. Peripheral blood samples from each subject were drawn into lithium heparin tubes, diluted 2-fold with PBS, and dispensed into a tissue culture plate. Cell wall antigens containing 5 μg of protein were added to the wells, and PBS was used as the “unstimulated” control. The plates were incubated at 37°C in 5% CO_2_ for 20 hours. After incubation, the plasma was separated from the cells and stored at 4°C until chitinase activity analysis.

### Statistics

*P*-values for pairwise comparisions were by Mann-Whitney *U* with Bonferroni adjustments for multiple comparisons. Global tests were by Kruskal-Wallis ANOVA. Surival curves were compared with Mann-Whitney tests. Power calculations were performed to assess appropriate sample size for all experiments. *P*-values ≤ 0.05 were considered statistically significant. All statistics and graphs were processed with Prism 6 (GraphPad Software, La Jolla, CA).

## Supporting Information

S1 Fig Helper T cell flow cytometry gating strategy. Single cell suspension isolated from lungs of wildtype mice 14 days post-infection with strain KN99α. Red or blue gate contains the entirety of the the subsequent plot below. “Th2” gate is drawn based on sample not incubated with PMA + ionomycin.
(TIF)

S2 Fig Bulk leukocyte flow cytometry gating strategy. Single cell suspension isolated from lungs of wildtype mice 14 days post-infection with strain KN99α. Red arrows and words indicate leukocyte subset.
(TIF)

S3 Fig Eosinophils predominate in the pulmonary leukocyte response to cryptococcal infection. Leukocytes harvested from (**A**) lungs or (**B**) mediastinal lymph nodes of mice 14 days post-infection with KN99α. Each subset is identified by non-redundant gating per S2 Fig.
(TIF)

S4 Fig Foxp3+ regulatory T cells specifically suppress type-2 helper T cells during pulmonary fungal infection. (**A**) Proportion of Th cells that express Foxp3 in wildtype mice infected and treated with IL-2, IL-2 antibody (JES6-1A12), or IL-2 complex. (**B-C**) Foxp3-*Diptheria* Toxin (DT) Receptor mice received DT every other day beginning at 5 days post-infection. Single cell suspensions isolated from lungs of wildtype and Foxp3-DTR mice at 14 days post-infection with KN99α were analyzed as the proportion of CD4+ cells expressing Foxp3 to monitor Treg depletion (**B**), or CD4+, Foxp3−, CD44+ Cda2+ Th cells expressing Th1 (IFNγ), Th2 (IL-5 & IL-13), or Th17 (IL-17A) cytokines to determine effector T cell differentiaion (**C**). Data are presented as mean ± standard error of the mean. * P < 0.05 and ***P < 0.0005 by Mann-Whitney *U*.
(TIF)

S5 Fig Analysis of CD11c+ cell subsets. **(A)** Diagram depicting the relationship between various CD11c+ cell subsets and mice used to delete/inhibit the subsets [5]. (**B**) Flow cytometry gating gtrategy. Single cell suspension isolated from lungs of wildtype or mutant mice 14 days post-infection with strain KN99α.
(TIF)

S6 Fig Macrophages, granulocytes, CD103+ conventional dendritic cells, monocytes, and monocyte-derived dendritic cells are dispensable for Th2 cell priming. **(A)** Eosinophils and neutrophils do not express MHCII during Cryptococcal infection. MHCII expression in eosinophils or neutrophils from lungs of mice 14 days post-infection with strain KN99α. Isotype refers to similar cells stained with a rat IgG2b antibody of irrelevant specificity. (**B-G**) Cells from wild-type, LysM-cre MHC fl/fl, CCR2−/−, or BATF3−/− mice 14 days post-infection with KN99α. (**B**) Representivive biexponential flow cytometry plot indicating the loss of macrophages in the lungs of LysM-cre MHCII fl/fl mice. (**C**) Quantification of antigen-specific Th2 cells in LysM-cre MHCII fl/fl infected mice. (**D**) Representative biexponential flow cytometry plot indicating the loss of monocytes (Ly6C+, CD11b+) and monocyte-derived DC (CD64+, CD11c+, MHCII+, FcεRI+) in the lungs of CCR2 −/− mice. (**E**) Representative biexponential flow cytometry plot indicating the loss of CD103+ conventional dendritic cells in BATF3−/− mice. (**F**) Quantification of monocytes and dendritic cell subsets from the lungs of mutant or wildtype mice. (**G**) Quantification of antigen-specific Th2 cells in wild-type, CCR2−/−, and BATF3−/− infected mice.
(TIF)

S7 Fig Cryptococcal chitin promotes polyclonal type-2 helper T cell accumulation. **(A)** Cryptococcal cells isolated from lungs at 14 days post-infection. Cryptococcal cells stained with Calcofluor White, and analyzed with flow cytometry. **(B)** CD4+ Foxp3- IL-5+ IL-13+ polyclonal Th2 cells in the lungs of mice infected with high chitin (KN99α, *rim101Δ*) or low chitin (*gpr4Δgpr5Δ*) cryptococcal strains. Data are presented as the mean +/− standard error with at least 2 independent experiments per group. *** = *P* < 0.0005 by Mann-Whitney *U*.
(TIF)

S8 Fig Chitotriosidase promotes Th2 cell accumulation without altering fungal burden. (**A**) Fungal burden in the lungs of wildtype, Chit1−/−, and AMCase−/− mice 14 days post-infection. (**B**) IL-5+ IL-13+ antigen-specific Th2 cells from lungs of mice 14 days post-infection. Data are presented as the mean +/- standard error with at least 2 independent experiments per group. ** = *P* < 0.005, *** = *P* < 0.0005 by Mann-Whitney *U* or Kruskal Wallis ANOVA.
(TIF)

S9 Fig CD11b+ conventional dendritic cells respond to chitin indirectly. Pulmonary leukocytes from wildtype mice 14 days post-infection with KN99 α. **(A)** CD19 (B cells) or CD11c (dendritic cells) co-labeled with R-phycoerithrin conjugated to streptavidin with biotinylated chitin heptamers (RPE-GN7) or without biotinylated chitin heptamers (RPE-SA). (**B**) IL-4 expression of CD11b+ conventional dendritic cells after 5 hours of stimulation with PMA + ionomycin, 125 μg of chitin heptamers (GN7) or left unstimulated. Histogram (**C**) and quantification of alarmin receptor expression (**D**) by CD11b+ conventional dendritic cells. Data are presented as the mean +/- standard error with at least 2 independent experiments per group. ** = *P* < 0.005, *** = *P* < 0.0005 by Mann-Whitney *U*. gMFI = geometric mean fluorescence intesity, TSLP = thymic stromal lymphopoietin.
(TIF)

S10 Fig *Cryptococcus* antigens and culture supernatants do not possess chitinase activity. Chitinase activity at pH = 2 and pH = 5 as measured in *Cryptococcus* lysate antigens and YPD supernatant from overnight cultures.
(TIF)

S1 Table Human demographic and clinical parameters. (DOCX)

S2 Table Summary of mice used. (DOCX)
