# Supplementary figures and images for: Chitin Recognition via Chitotriosidase Promotes Pathologic Type-2 Helper T Cell Responses to Cryptococcal Infection
Source: PLoS Pathog. 2015 Mar 12;11(3):e1004701. doi: 10.1371/journal.ppat.1004701 (PMC4357429; doi:10.1371/journal.ppat.1004701)

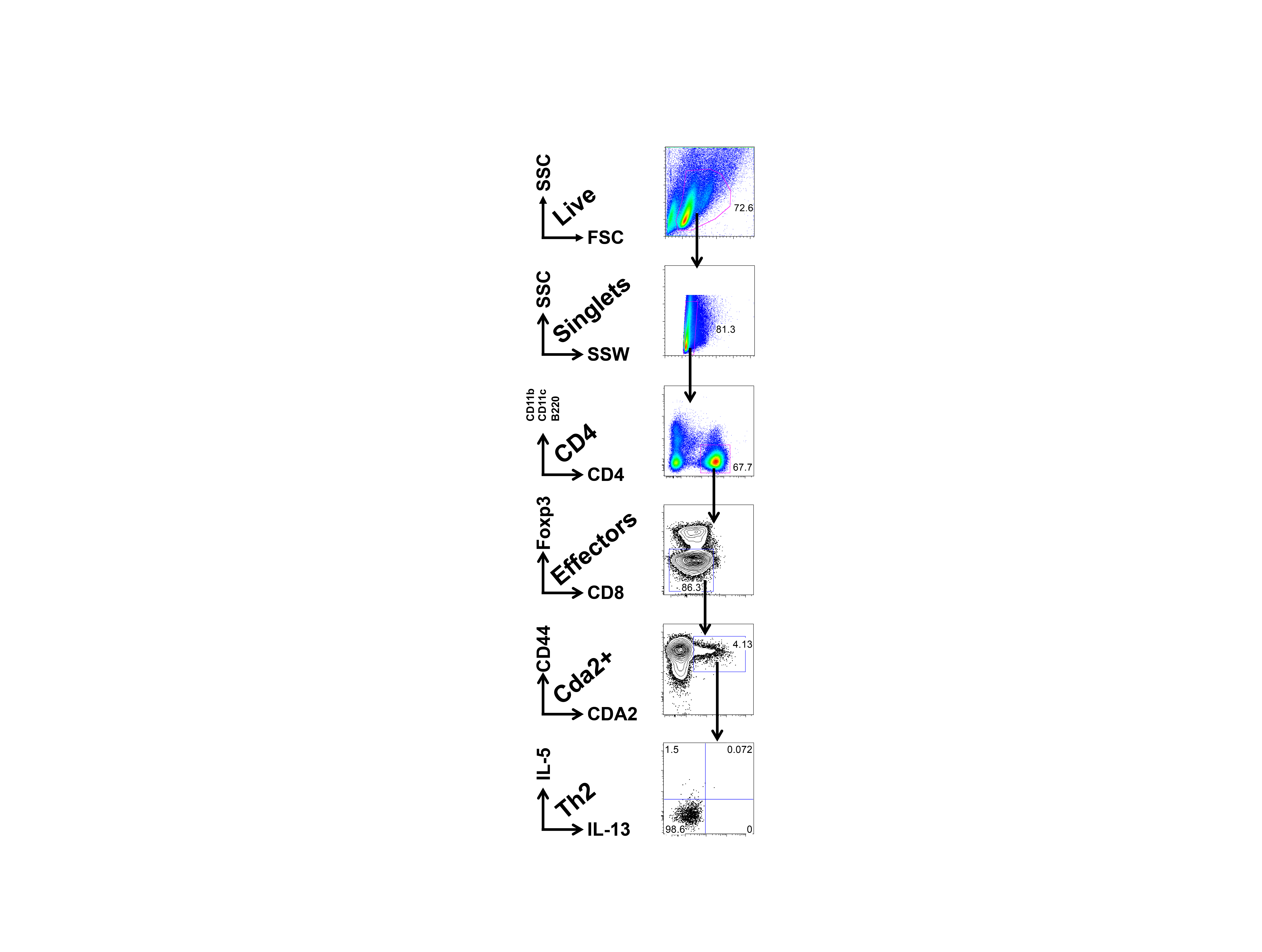

Supplement: S1 Fig — Single cell suspension isolated from lungs of wildtype mice 14 days post-infection with strain KN99α. Red or blue gate contains the entirety of the the subsequent plot below. “Th2” gate is drawn based on sample not incubated with PMA + ionomycin. (TIF) [file ppat.1004701.s001.tif]

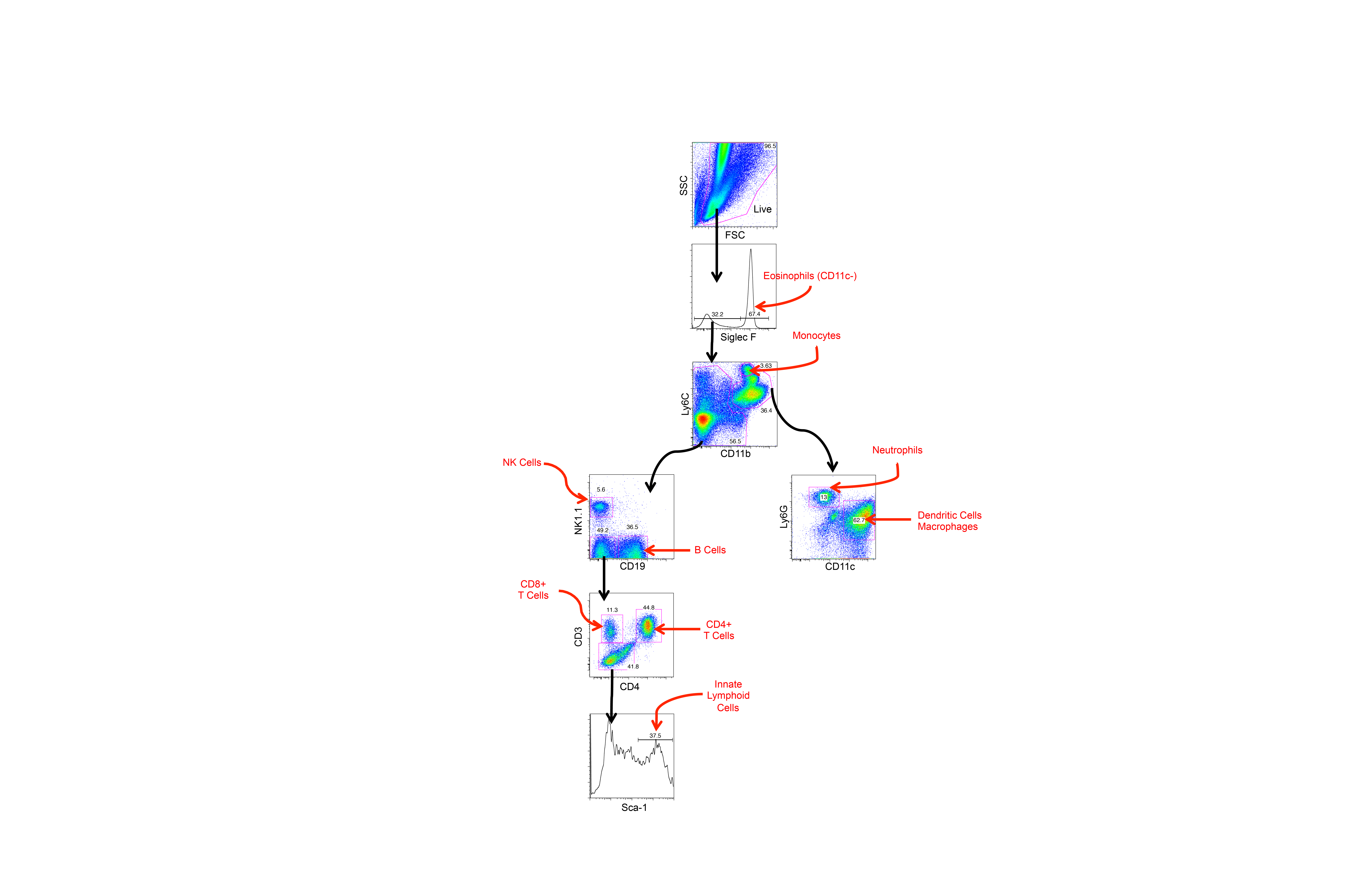

Supplement: S2 Fig — Single cell suspension isolated from lungs of wildtype mice 14 days post-infection with strain KN99α. Red arrows and words indicate leukocyte subset. (TIF) [file ppat.1004701.s002.tif]

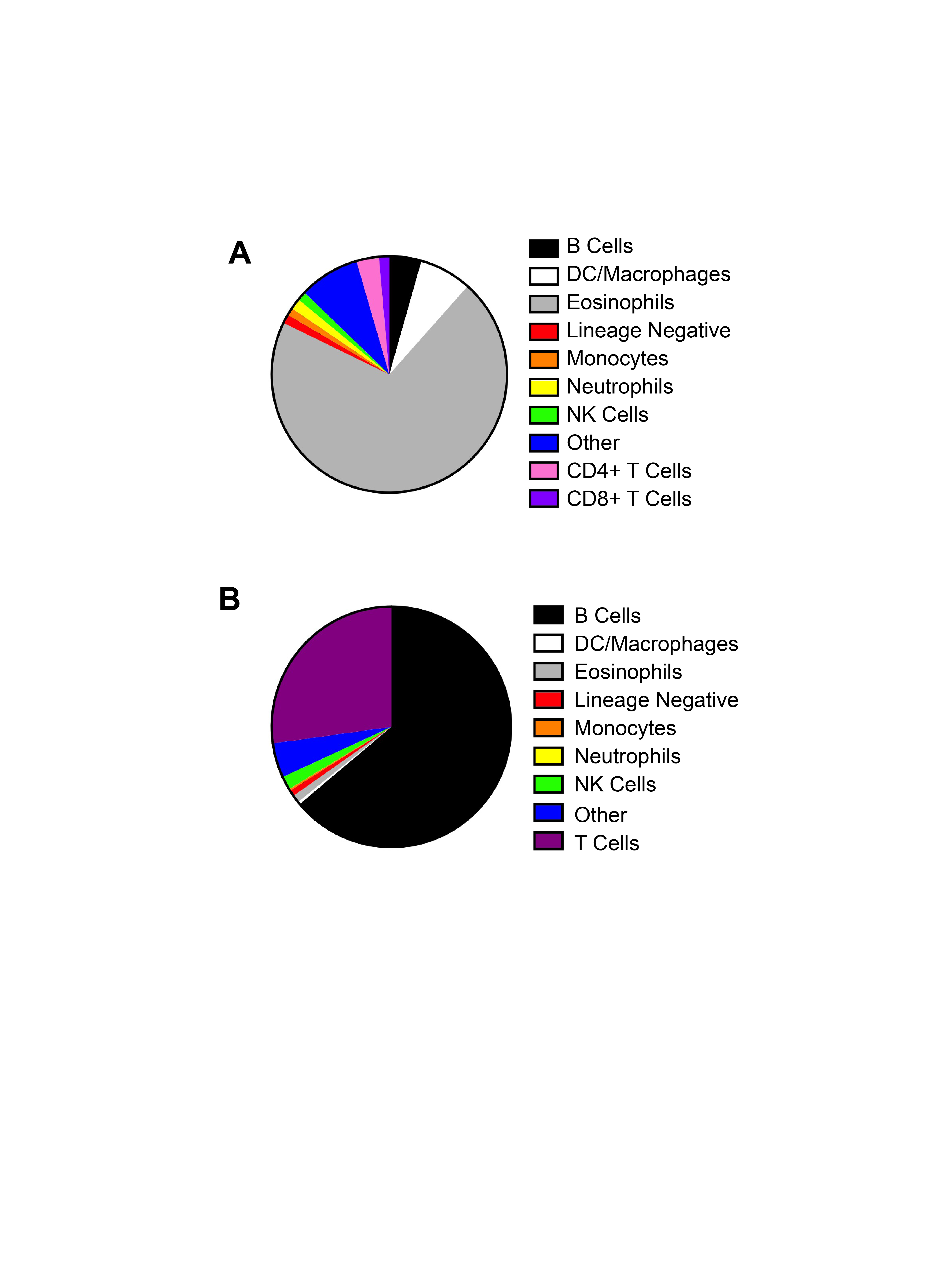

Supplement: S3 Fig — Leukocytes harvested from (A) lungs or (B) mediastinal lymph nodes of mice 14 days post-infection with KN99α. Each subset is identified by non-redundant gating per S2 Fig. (TIF) [file ppat.1004701.s003.tif]

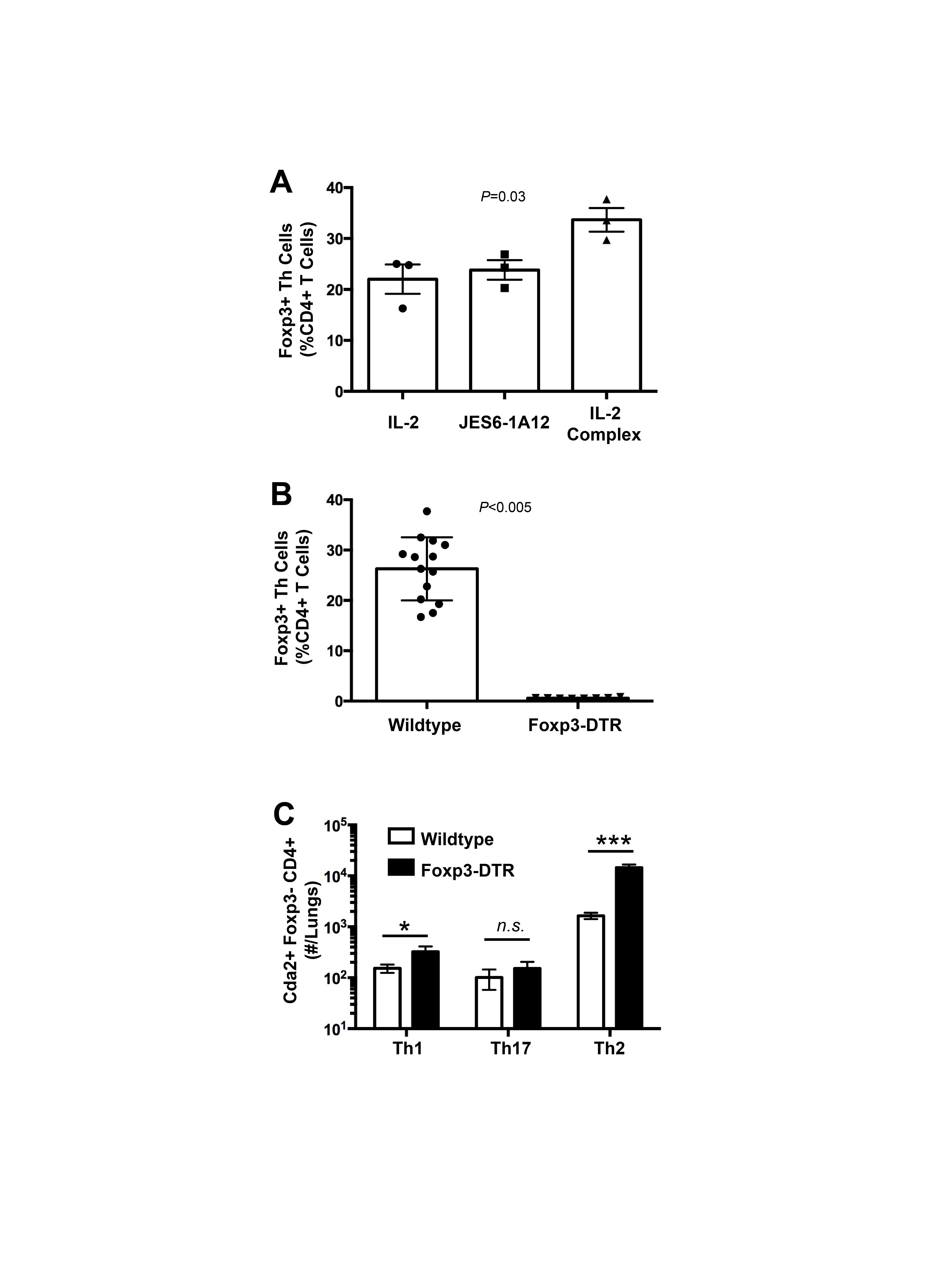

Supplement: S4 Fig — (A) Proportion of Th cells that express Foxp3 in wildtype mice infected and treated with IL-2, IL-2 antibody (JES6-1A12), or IL-2 complex. (B-C) Foxp3-Diptheria Toxin (DT) Receptor mice received DT every other day beginning at 5 days post-infection. Single cell suspensions isolated from lungs of wildtype and Foxp3-DTR mice at 14 days post-infection with KN99α were analyzed as the proportion of CD4+ cells expressing Foxp3 to monitor Treg depletion (B), or CD4+, Foxp3−, CD44+ Cda2+ Th cells expressing Th1 (IFNγ), Th2 (IL-5 & IL-13), or Th17 (IL-17A) cytokines to determine effector T cell differentiaion (C). Data are presented as mean ± standard error of the mean. * P < 0.05 and ***P < 0.0005 by Mann-Whitney U. (TIF) [file ppat.1004701.s004.tif]

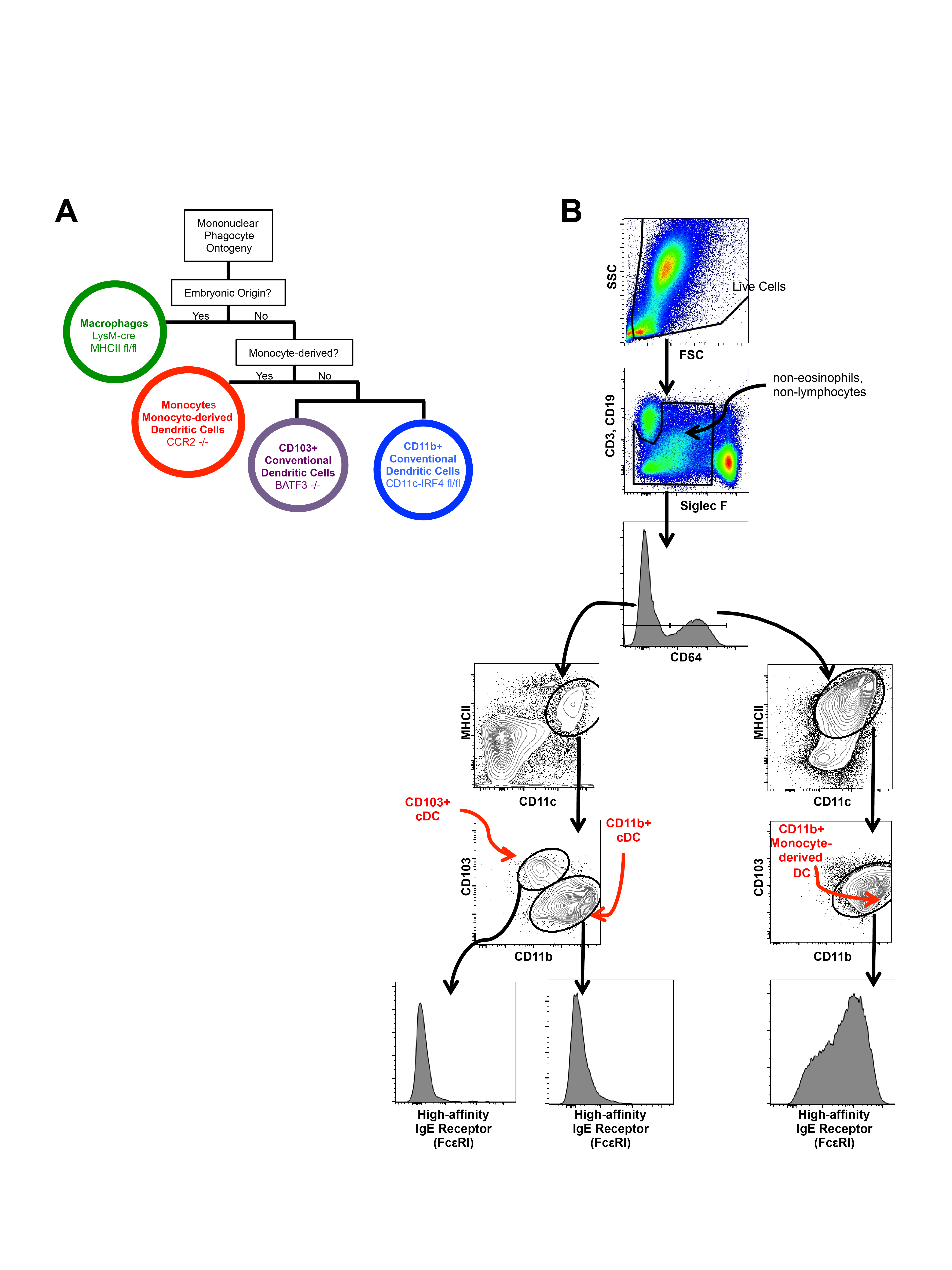

Supplement: S5 Fig — (A) Diagram depicting the relationship between various CD11c+ cell subsets and mice used to delete/inhibit the subsets [5]. (B) Flow cytometry gating gtrategy. Single cell suspension isolated from lungs of wildtype or mutant mice 14 days post-infection with strain KN99α. (TIF) [file ppat.1004701.s005.tif]

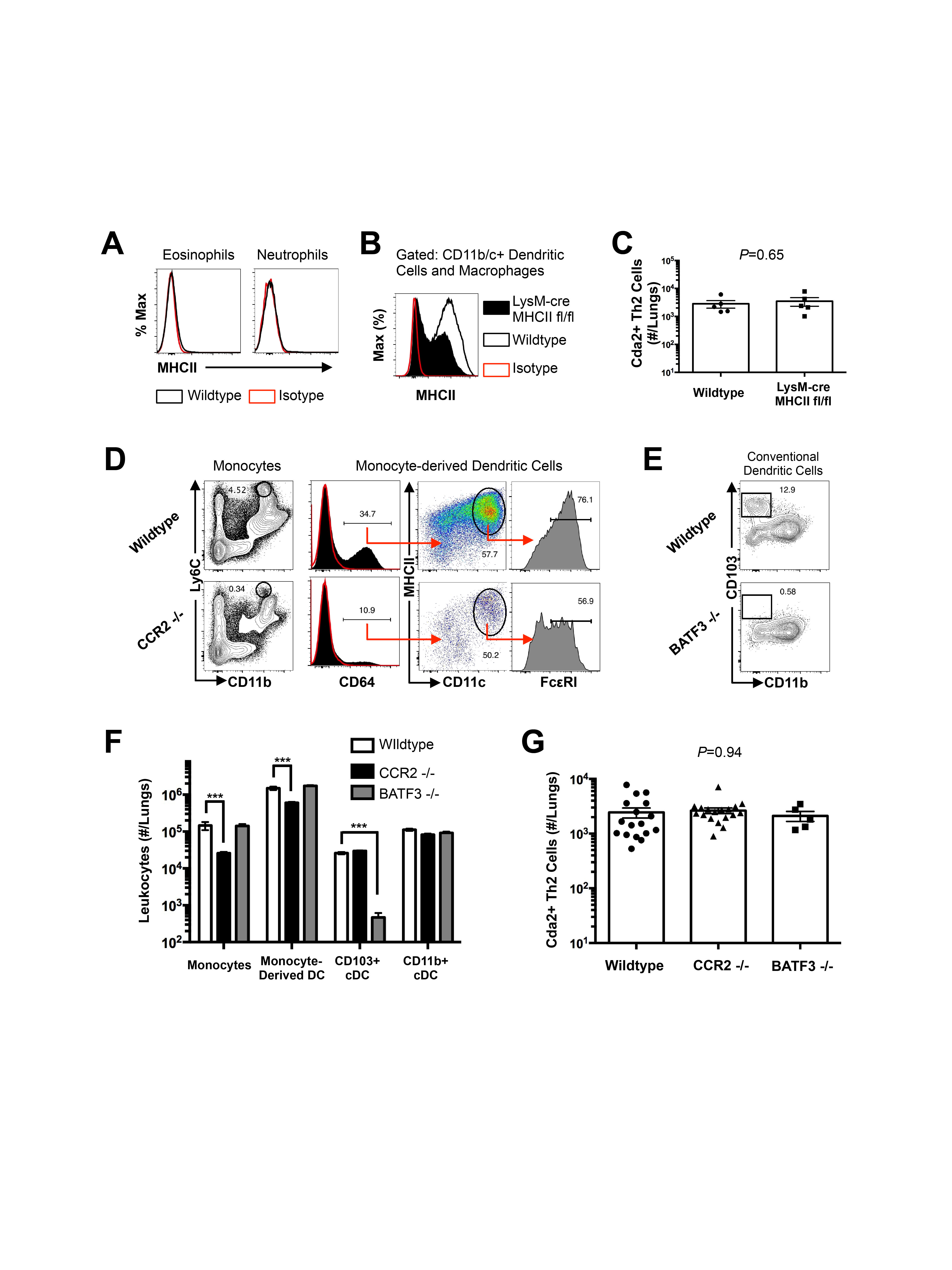

Supplement: S6 Fig — (A) Eosinophils and neutrophils do not express MHCII during Cryptococcal infection. MHCII expression in eosinophils or neutrophils from lungs of mice 14 days post-infection with strain KN99α. Isotype refers to similar cells stained with a rat IgG2b antibody of irrelevant specificity. (B-G) Cells from wild-type, LysM-cre MHC fl/fl, CCR2−/−, or BATF3−/− mice 14 days post-infection with KN99α. (B) Representivive biexponential flow cytometry plot indicating the loss of macrophages in the lungs of LysM-cre MHCII fl/fl mice. (C) Quantification of antigen-specific Th2 cells in LysM-cre MHCII fl/fl infected mice. (D) Representative biexponential flow cytometry plot indicating the loss of monocytes (Ly6C+, CD11b+) and monocyte-derived DC (CD64+, CD11c+, MHCII+, FcεRI+) in the lungs of CCR2 −/− mice. (E) Representative biexponential flow cytometry plot indicating the loss of CD103+ conventional dendritic cells in BATF3−/− mice. (F) Quantification of monocytes and dendritic cell subsets from the lungs of mutant or wildtype mice. (G) Quantification of antigen-specific Th2 cells in wild-type, CCR2−/−, and BATF3−/− infected mice. (TIF) [file ppat.1004701.s006.tif]

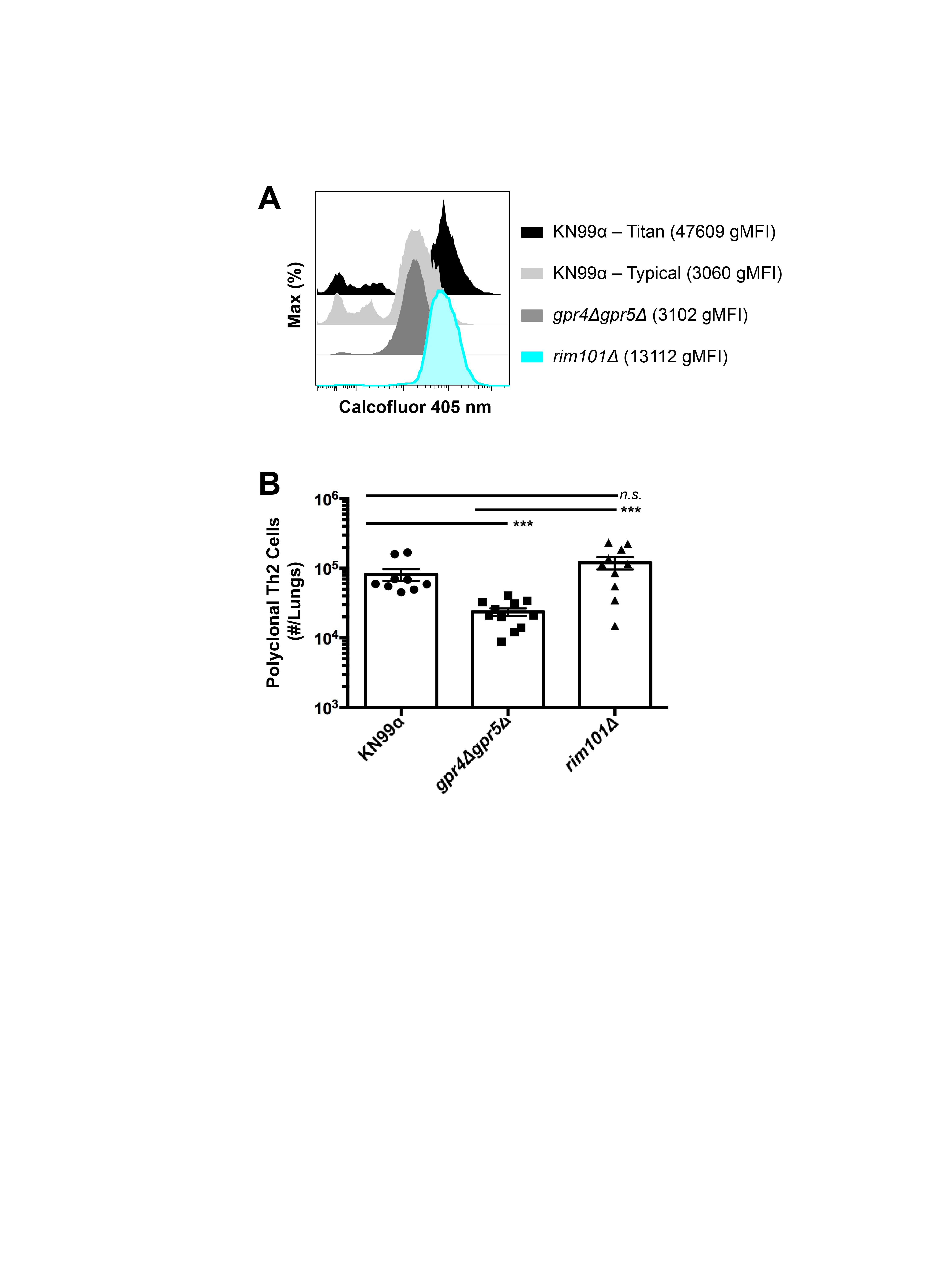

Supplement: S7 Fig — (A) Cryptococcal cells isolated from lungs at 14 days post-infection. Cryptococcal cells stained with Calcofluor White, and analyzed with flow cytometry. (B) CD4+ Foxp3- IL-5+ IL-13+ polyclonal Th2 cells in the lungs of mice infected with high chitin (KN99α, rim101Δ) or low chitin (gpr4Δgpr5Δ) cryptococcal strains. Data are presented as the mean +/− standard error with at least 2 independent experiments per group. *** = P < 0.0005 by Mann-Whitney U. (TIF) [file ppat.1004701.s007.tif]

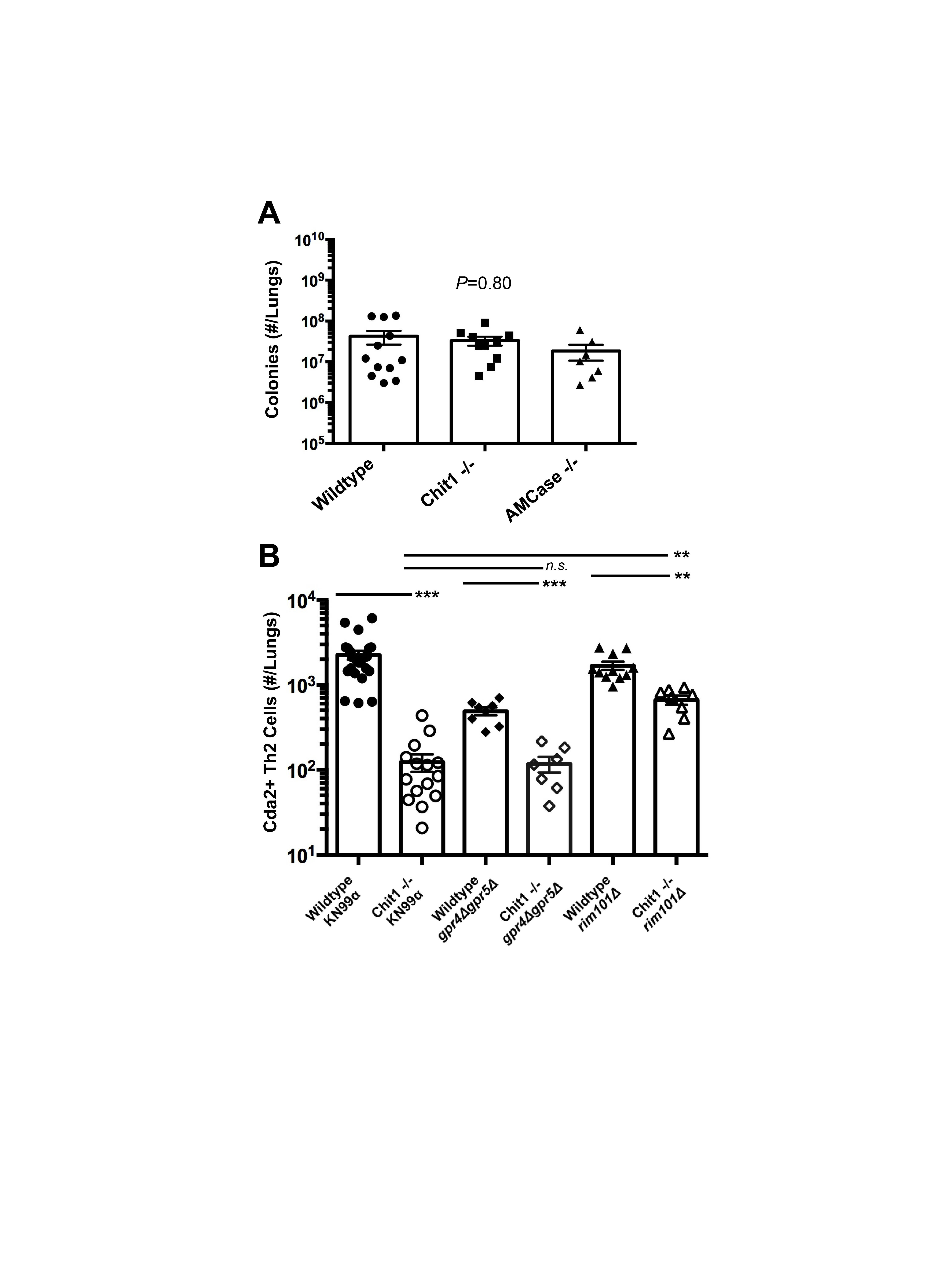

Supplement: S8 Fig — (A) Fungal burden in the lungs of wildtype, Chit1−/−, and AMCase−/− mice 14 days post-infection. (B) IL-5+ IL-13+ antigen-specific Th2 cells from lungs of mice 14 days post-infection. Data are presented as the mean +/- standard error with at least 2 independent experiments per group. ** = P < 0.005, *** = P < 0.0005 by Mann-Whitney U or Kruskal Wallis ANOVA. (TIF) [file ppat.1004701.s008.tif]

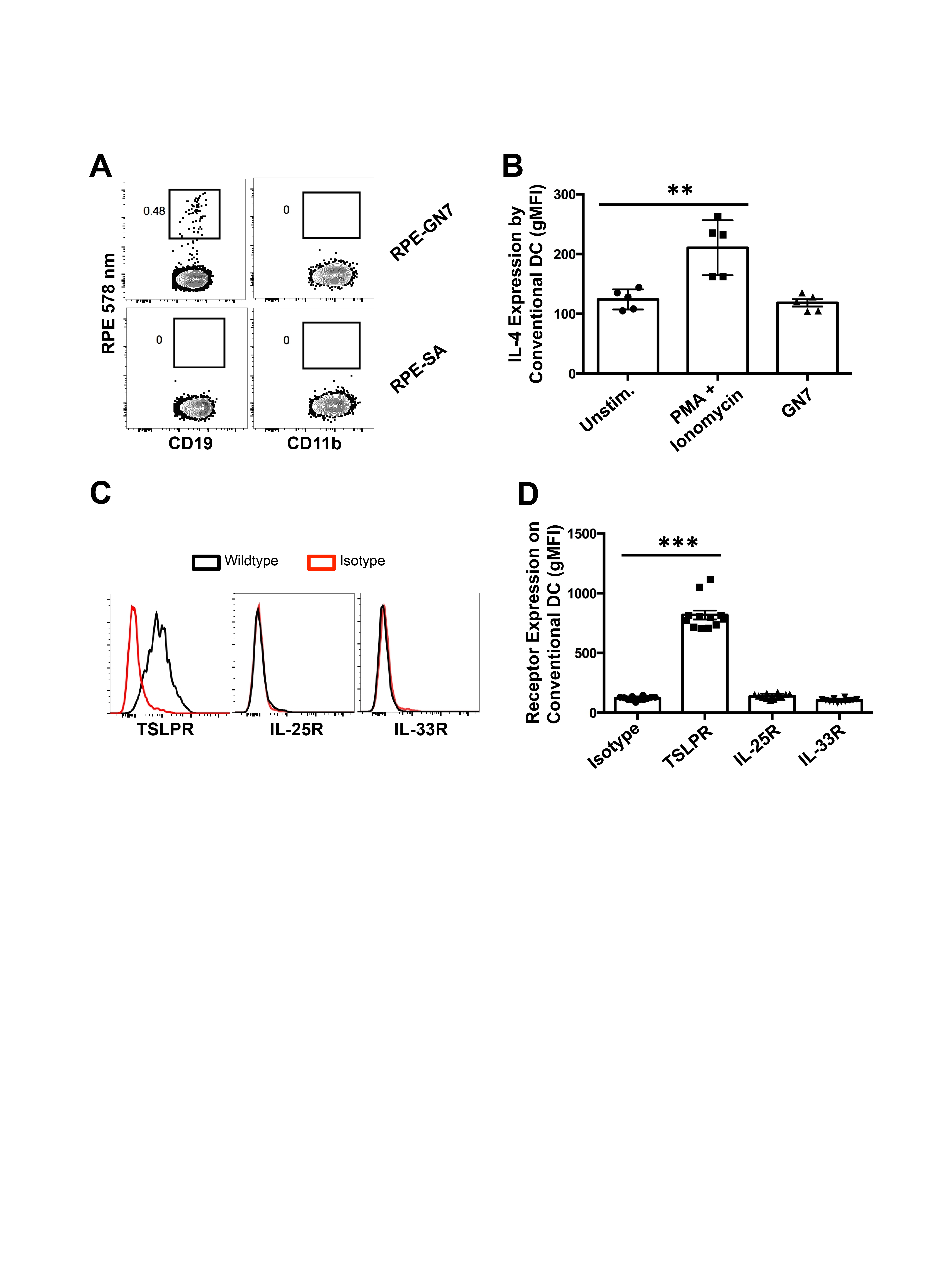

Supplement: S9 Fig — Pulmonary leukocytes from wildtype mice 14 days post-infection with KN99 α. (A) CD19 (B cells) or CD11c (dendritic cells) co-labeled with R-phycoerithrin conjugated to streptavidin with biotinylated chitin heptamers (RPE-GN7) or without biotinylated chitin heptamers (RPE-SA). (B) IL-4 expression of CD11b+ conventional dendritic cells after 5 hours of stimulation with PMA + ionomycin, 125 μg of chitin heptamers (GN7) or left unstimulated. Histogram (C) and quantification of alarmin receptor expression (D) by CD11b+ conventional dendritic cells. Data are presented as the mean +/- standard error with at least 2 independent experiments per group. ** = P < 0.005, *** = P < 0.0005 by Mann-Whitney U. gMFI = geometric mean fluorescence intesity, TSLP = thymic stromal lymphopoietin. (TIF) [file ppat.1004701.s009.tif]

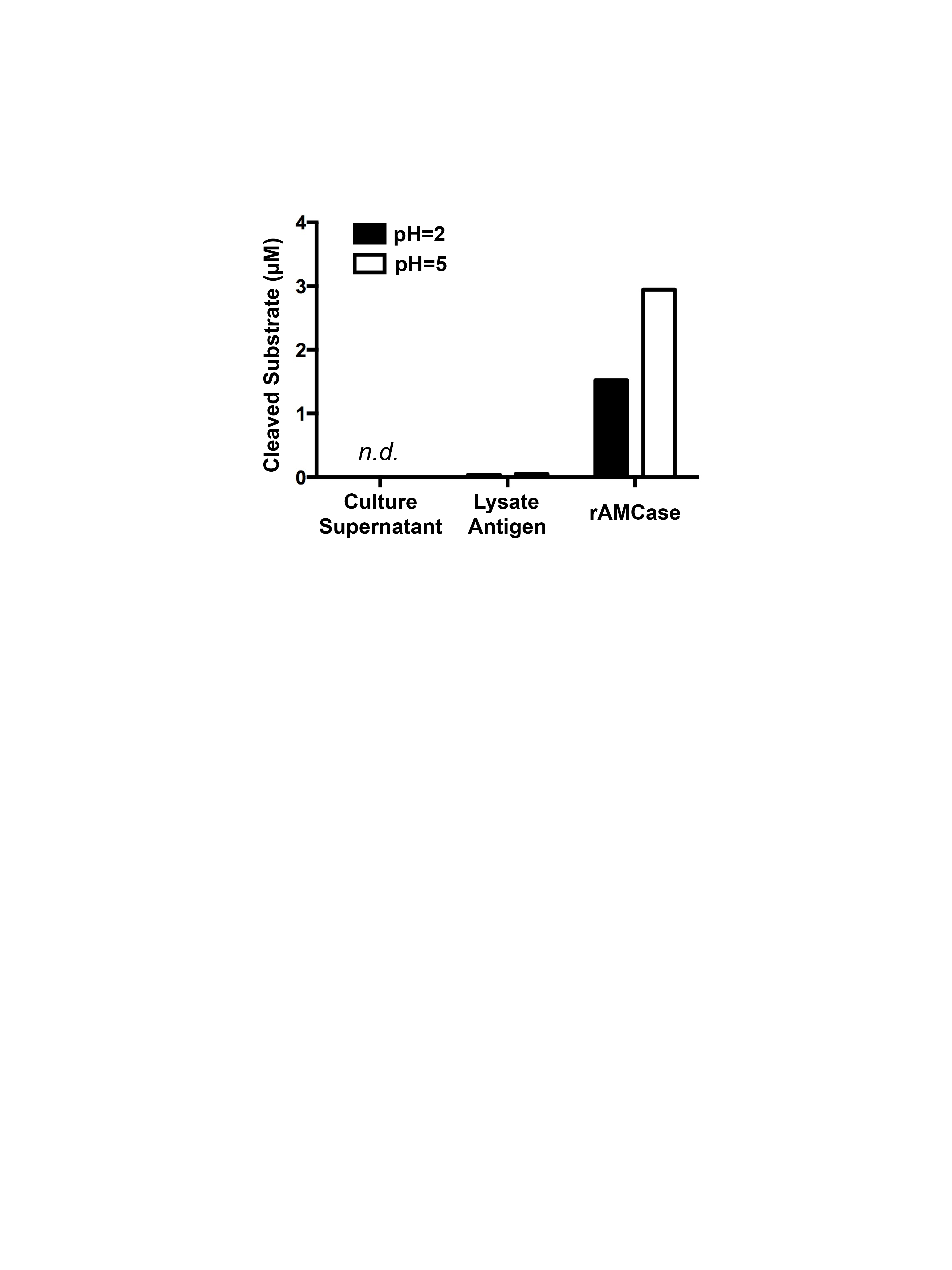

Supplement: S10 Fig — Chitinase activity at pH = 2 and pH = 5 as measured in Cryptococcus lysate antigens and YPD supernatant from overnight cultures. (TIF) [file ppat.1004701.s010.tif]
