# Supplementary material for: Chitin Recognition via Chitotriosidase Promotes Pathologic Type-2 Helper T Cell Responses to Cryptococcal Infection
Source: PLoS Pathog. 2015 Mar 12;11(3):e1004701. doi: 10.1371/journal.ppat.1004701 (PMC4357429; doi:10.1371/journal.ppat.1004701)
Supplement: S1 Table — (DOCX) [file ppat.1004701.s011.docx]

**Supplemental Table 1. Human Demographic and Clinical Parameters**

|  | **AIDS^a^** | **AIDS + *Cryptococcus*** | ***P*-value** |
| --- | --- | --- | --- |
| **Sex (M/F)** | 25/21 | 19/19 | 0.69 |
| **Age (years)** | 34.0 +/- 0.99 | 35.7 +/- 1.19 | 0.28 |
| **WBC (10^3^ cells/µL Blood)** | 5.15 +/- 0.48 | 4.00 +/- 0.35 | 0.07 |
| **Lymphocytes (10^3^ cells/µL Blood)** | 1.46 +/- 0.26 | 0.95 +/- 0.12 | 0.10 |

^a^ Disease etiology: viral meningitis (7), tubercular meningitis (4), meningococcal meningitis (7), or unknown diagnosis (29).
