# Supplementary material for: Chitin Recognition via Chitotriosidase Promotes Pathologic Type-2 Helper T Cell Responses to Cryptococcal Infection
Source: PLoS Pathog. 2015 Mar 12;11(3):e1004701. doi: 10.1371/journal.ppat.1004701 (PMC4357429; doi:10.1371/journal.ppat.1004701)
Supplement: S2 Table — (DOCX) [file ppat.1004701.s012.docx]

**Supplemental Table 2. Summary of Mice Used**

| **Mouse Strain** | **Phenotype** | **Citation** |
| --- | --- | --- |
| **CD11c-cre +/0 MHCII fl/fl** | Deletion of MHCII in dendritic cells | [1] |
| **CD11c-cre +/0 IRF4 fl/fl** | Loss of CD11b+ conventional dendritic cell maturation | [2-5] |
| **LysM-cre +/- MHCII fl/fl** | Deletion of MHCII in macrophages | This study |
| **CCR2 -/-** | Loss of monocytes and monocyte-derived dendritic cells | [6] |
| **Flt3L -/-** | Abolishes mononuclear phagocyte traffic to lymph nodes | [7] |
| **AMCase -/-** | Global knockout of acidic mammalian chitinase | [8] |
| **Chit1 -/-** | Global knockout of chitotriosidase | [9] |
| **Batf3 -/-** | Developmental blockade in CD103+ conventional dendritic cells | [10] |
| **Foxp3-DTR** | Inducible depletion of regulatory T cells upon *Diphtheria* toxin administration | [11] |

1. Darrasse-Jeze G, Deroubaix S, Mouquet H, Victora GD, Eisenreich T, et al. (2009) Feedback control of regulatory T cell homeostasis by dendritic cells in vivo. J Exp Med 206: 1853-1862.

2. Williams JW, Tjota MY, Clay BS, Vander Lugt B, Bandukwala HS, et al. (2013) Transcription factor IRF4 drives dendritic cells to promote Th2 differentiation. Nat Commun 4: 2990.

3. Gao Y, Nish SA, Jiang R, Hou L, Licona-Limon P, et al. (2013) Control of T helper 2 responses by transcription factor IRF4-dependent dendritic cells. Immunity 39: 722-732.

4. Schlitzer A, McGovern N, Teo P, Zelante T, Atarashi K, et al. (2013) IRF4 transcription factor-dependent CD11b+ dendritic cells in human and mouse control mucosal IL-17 cytokine responses. Immunity 38: 970-983.

5. Persson EK, Uronen-Hansson H, Semmrich M, Rivollier A, Hagerbrand K, et al. (2013) IRF4 transcription-factor-dependent CD103(+)CD11b(+) dendritic cells drive mucosal T helper 17 cell differentiation. Immunity 38: 958-969.

6. Boring L, Gosling J, Chensue SW, Kunkel SL, Farese RV, Jr., et al. (1997) Impaired monocyte migration and reduced type 1 (Th1) cytokine responses in C-C chemokine receptor 2 knockout mice. J Clin Invest 100: 2552-2561.

7. McKenna HJ, Stocking KL, Miller RE, Brasel K, De Smedt T, et al. (2000) Mice lacking flt3 ligand have deficient hematopoiesis affecting hematopoietic progenitor cells, dendritic cells, and natural killer cells. Blood 95: 3489-3497.

8. Fitz LJ, DeClercq C, Brooks J, Kuang W, Bates B, et al. (2012) Acidic mammalian chitinase is not a critical target for allergic airway disease. Am J Respir Cell Mol Biol 46: 71-79.

9. Lee CG, Herzog EL, Ahangari F, Zhou Y, Gulati M, et al. (2012) Chitinase 1 is a biomarker for and therapeutic target in scleroderma-associated interstitial lung disease that augments TGF-beta1 signaling. J Immunol 189: 2635-2644.

10. Hildner K, Edelson BT, Purtha WE, Diamond M, Matsushita H, et al. (2008) Batf3 deficiency reveals a critical role for CD8alpha+ dendritic cells in cytotoxic T cell immunity. Science 322: 1097-1100.

11. Kim JM, Rasmussen JP, Rudensky AY (2007) Regulatory T cells prevent catastrophic autoimmunity throughout the lifespan of mice. Nat Immunol 8: 191-197.
